# Applying Physics-Based Scoring to Calculate Free Energies of Binding for Single Amino Acid Mutations in Protein-Protein Complexes

**DOI:** 10.1371/journal.pone.0082849

**Published:** 2013-12-10

**Authors:** Hege Beard, Anuradha Cholleti, David Pearlman, Woody Sherman, Kathryn A. Loving

**Affiliations:** 1 Schrödinger, New York, New York, United States of America; 2 Schrödinger, Hyderabad, Andhra Pradesh, India; Wake Forest University, United States of America

## Abstract

Predicting changes in protein binding affinity due to single amino acid mutations helps us better understand the driving forces underlying protein-protein interactions and design improved biotherapeutics. Here, we use the MM-GBSA approach with the OPLS2005 force field and the VSGB2.0 solvent model to calculate differences in binding free energy between wild type and mutant proteins. Crucially, we made no changes to the scoring model as part of this work on protein-protein binding affinity—the energy model has been developed for structure prediction and has previously been validated only for calculating the energetics of small molecule binding. Here, we compare predictions to experimental data for a set of 418 single residue mutations in 21 targets and find that the MM-GBSA model, on average, performs well at scoring these single protein residue mutations. Correlation between the predicted and experimental change in binding affinity is statistically significant and the model performs well at picking “hotspots,” or mutations that change binding affinity by more than 1 kcal/mol. The promising performance of this physics-based method with no tuned parameters for predicting binding energies suggests that it can be transferred to other protein engineering problems.

## Introduction

Proteins can be modified to serve multiple different functional roles, with practical applications in medicine, industry, and basic science. Protein engineers use a combination of computational approaches and experimental techniques to find sequence variations that can modulate protein function. Computational protein design has yielded impressive successes, such as predicting a completely novel protein fold [1], designing a zinc-finger protein that folds without a metal cofactor [2], introducing catalytic activity into a protein that is not an enzyme [3], and creating an enzyme for which there is no naturally occurring biocatalyst [4]. However, the overall success rate is low for *de novo* computational enzyme design, due to the extremely challenging nature of the problem [5], and successful attempts to design higher affinity or stability proteins have often used an iterative approach where single mutations are validated separately and then combined [6,7,8,9].

While the above noted design successes are impressive, most practitioners take a more conservative approach to computational protein design, either by designing large protein libraries or by making single residue changes to modulate existing function. Protein library design involves suggesting specific sequences to be made experimentally, where computation is used to focus experiments on the parts of sequence space that are most likely to contain the desired protein function [10,11,12]. On the other hand, rational design can be used to predict specific amino acid modifications — even a single mutation can significantly impact protein solubility, stability, or affinity — and this may be the best approach to a given design problem.

Various computational methods have been used to predict the impact of single amino acid mutations, and in particular these methods are successful at distinguishing protein “hotspots” (positions where mutations have a large impact on protein stability or affinity) from other positions where mutations have little to no impact on these properties [13,14,15,16]. Predicting both types of mutations can be useful because even when a single mutation has no direct impact on the protein function, it may modulate pharmacokinetic and ADME properties or increase the efficiency of manufacturing the protein. Computational methods can effectively be used to predict the impact of a large number of single mutations on various protein properties. Computational methods applied towards this purpose have included structure-based modeling of atomic interactions (electrostatics, packing, solvation, etc.) as well as data mining in sequence space [3,17,18,19]. For example, a single histidine mutant was used to increase the half-life of granulocyte colony stimulating factor (GCSF) [20], and single mutations have been used to modulate protein-protein interaction specificity [21,22,23].

In this work, we present results for computational affinity predictions of single mutations at protein-protein interfaces using the MM-GBSA approach [24,25] in BioLuminate (version 1.0, Schrödinger, LLC, New York, NY, 2012), which incorporates the OPLS2005 force field [26,27], VSGB solvent model [28], and rotamer search algorithms from Prime (version 3.1, Schrödinger, LLC, New York, NY, 2012). For our test set we used the proteins and mutations from Kortemme and Baker [13], since this is a well-known dataset in the protein design field. Importantly, we did not use any training set to modify our force field or solvent model during this work. Furthermore, the Prime sampling algorithm and solvent model have been fit to reproduce side-chain conformations in protein crystal structures [29,30], not binding free energies. We present the results for the computational method described in this work and highlight important considerations when predicting protein-protein binding energies, such as careful protein preparation and consideration of protein stability during computational protein design.

## Results and Discussion

### Performance on the 19 target data set

We performed MM-GBSA scoring of mutations for the 19 targets in the Kortemme and Baker test set [13], as described in the Materials and Methods. The MM-GBSA score for a protein-protein complex is calculated by scoring three protein structures (one binding partner alone, the other binding partner alone, and the complex) using the OPLS2005 force field and an implicit solvent model for water. By comparing the energies of these three systems for the wild type to the same three systems for the mutant, we determine a predicted change in binding affinity for changing the wild-type residue to the mutant. The equation for this “delta Affinity” is described in the Materials and Methods, and is derived from a well-known thermodynamic cycle.

**Figure 1:**
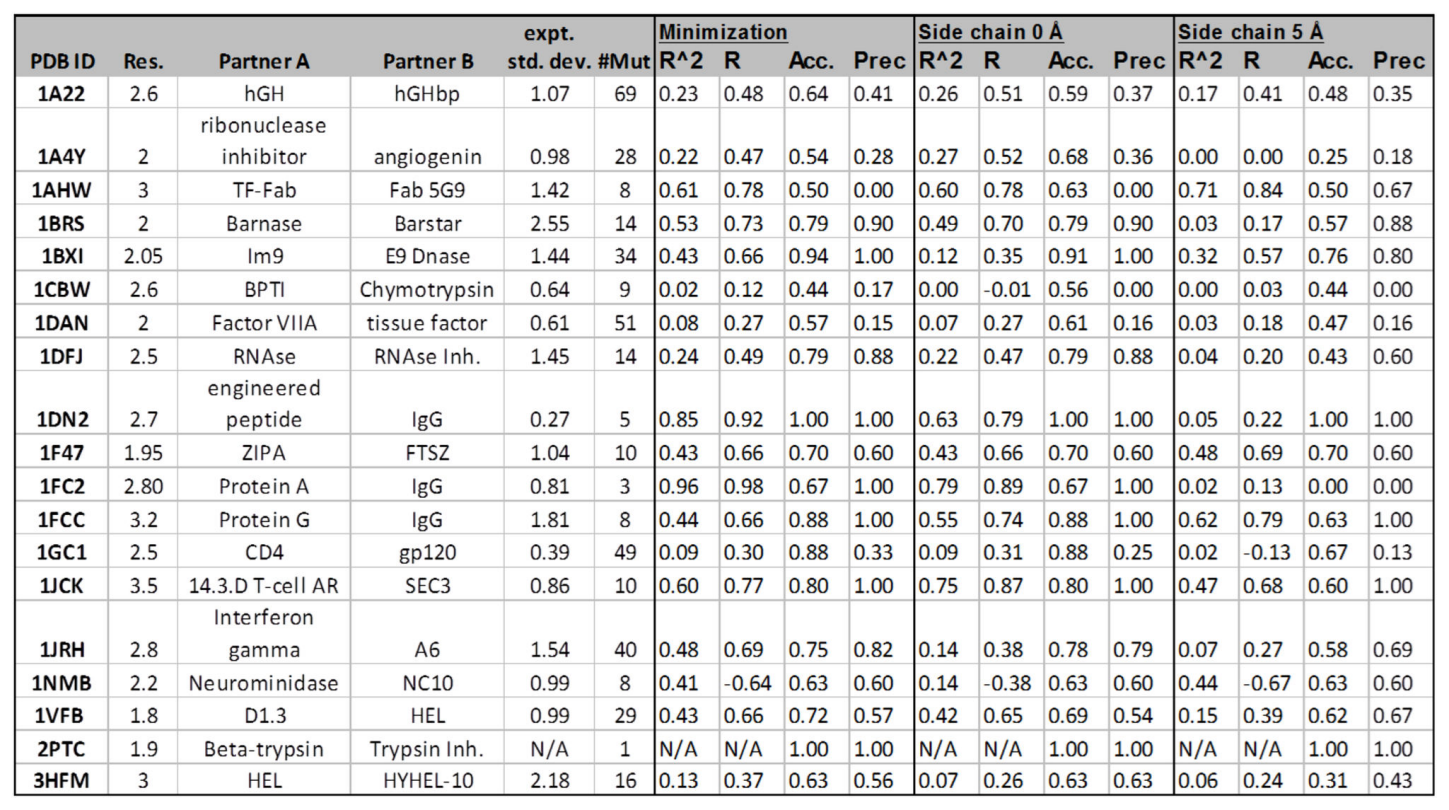
Results summary for predicted change in protein-protein binding affinity for 19 protein-protein interaction targets [13]. Each R^2^ is the correlation between the predicted change in binding affinity and the experimental change in binding affinity. Three different refinement methods were used, as described in Materials and Methods. Accuracy is an overall measure of the ability to categorize residues as “neutral” or “hot spot” (see Results and Discussion). “Hotspot precision” indicates the ability of the model to select mutations that make binding affinity worse by more than 1 kcal/mol. The experimental standard deviation is computed after removing qualifiers from qualified values (i.e. >2.0 is treated as 2.0) and has units of kcal/mol.

We tested varying degrees of protein flexibility, from minimization of the residue of interest alone (with the rest of the protein rigid) to side-chain sampling of the residue of interest plus all other residues in a radius of 5 Å. For most of the systems, a very conservative level of protein flexibility (i.e. minimization of only the mutated side chain of interest) produced the best correlations between the calculated and experimental results (Figure 1). The full set of experimental and predicted mutations is included as Table S1.

While correlation (r or R^2^) can be a useful assessment metric in some cases, it is not an optimal measure of performance for practical applications, where a key objective is to assess the ability of a method to prioritize mutations that improve affinity. To address the limitations associated with correlations, Kortemme and Baker followed up on Clackson and Wells’ concept of a “hotspot” [31] as a mutation that weakens binding affinity by more than 1 kcal/mol. In their paper they report a “fraction correct”, which is defined as the fraction of experimental hotspots that are computationally predicted to be hotspots. This metric has an intuitive interpretation and addresses some of the limitations associated with correlations, but it does not provide a complete assessment of a method because it combines all non-hotspot mutations into one category (those predicted to improve binding affinity by more than 1 kcal/mol and those predicted to be neutral).

In the work presented here, we use metrics that measure the ability of a method to correctly predict mutations that will have an impact (positive or negative) on binding. As such, we categorize mutations into “neutral” (experimentally within 1 kcal/mol of zero change in binding affinity) or “hotspot” (more than a 1 kcal/mol change in binding affinity). We include two different hotspot classes: at least 1 kcal/mol decrease in binding affinity, and at least 1 kcal/mol improvement in binding affinity, for a total of three mutation classes. We define “hotspot precision” in the same way as the traditional use of precision: the sum of true positive hotspot predictions divided by the total number of hotspot predictions (true positives plus false positives). In other words, out of the mutations computationally predicted as hotspots, what fraction are true experimental hotspots. This directly measures the ability to select true hotspots from a dataset. We also use an “accuracy” metric to describe the overall correctness of our model, defined as the sum of correct classifications divided by the total number of classifications [32]. Given these categories, the null model for hotspot precision and accuracy is 1/3, which corresponds to predicted and experimental values randomly distributed between the three classes.

As described in the Materials and Methods, the change in binding affinity calculated with MM-GBSA is on a different scale than the experimental change in binding affinity. Here, we use a cutoff of 3 kcal/mol to define the hotspot classes for the predicted affinities by MM-GBSA. This number reflects the typical slope of the line we observe when comparing computed to experimental binding energies with MM-GBSA for small molecule binding. While better results could be obtained in this work with a fit to the computed protein-protein binding energies, our intention was to avoid any fit parameters in this work. In addition to hotspot precision and accuracy, we also report the “fraction correct” metric in Figure S1, which shows that MM-GBSA has a slightly higher fraction correct for hotspots while Rosetta has a slightly higher fraction correct for neutral mutations. Overall, the methods perform about the same.

**Figure 2:**
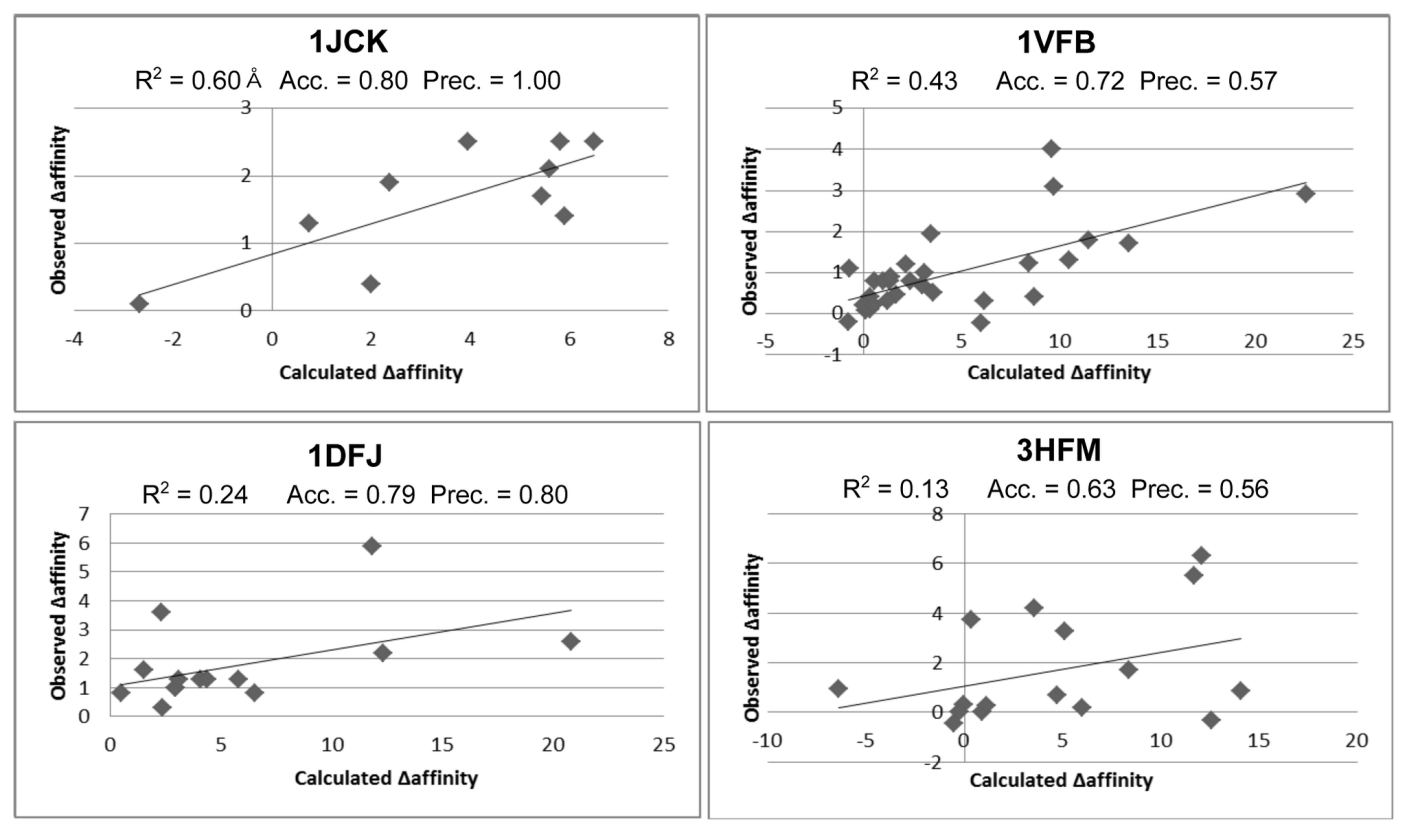
Plots of observed versus predicted affinity using the “minimization” refinement method, for four targets: 1JCK, 1VFB, 1DFJ, and 3HFM. The R^2^ correlation, accuracy, and hotspot precision are shown for each target.

Figure 2 shows the experimental and predicted changes in binding affinity for four targets with alanine mutational data studied here and illustrates differences in the model performance across different systems. For example, 1JCK and 1VFB have good correlation (R^2^ is 0.59 and 0.43, respectively) and the accuracy and precision metrics are also good. For 1JCK, we predict 4 mutations as neutral and 6 mutations as hotspots. These 6 hotspot predictions are all correct (experimentally they decrease binding affinity by more than 1 kcal/mol), resulting in a hotspot precision for this target of 1.0. However, two of the predicted neutral mutations are experimental hotspots, leading to an overall accuracy of 0.8. For 1VFB, we predict that 15 mutations will be neutral and 14 will be hotspots. 8 of the 14 predicted hotspots are true hotspots, so the hotspot precision for this target is 0.57. In addition, 13 of the 15 predicted neutral predictions are true neutral mutations and the hotspot classification accuracy for this target is 0.72. These system dependent differences do not appear to be due to the character of the mutated residues – in Table S2 we separated the mutations into groups by residue type and observed only a very small difference in the R^2^ correlation when the wild-type residue was hydrophobic (0.22), polar (0.39), or charged (0.26).

Figure 2 also highlights two targets (1DFJ and 3HFM) where the correlation between predicted and experimental changes in binding affinity are small (R^2^ is 0.24 and 0.13, respectively) but the accuracy and hotspot precision evaluation metrics are good. This shows that MM-GBSA can be useful for prediction of mutations as neutral, significantly improving binding, or making binding affinity significantly worse. Of the 14 mutations in 1DFJ, we predict 6 to be neutral and 8 to be hotspots. Indeed, 4 of the 6 neutral mutations are true neutral mutations, while 7 of the predicted hotspots are true hotspots, resulting in a hotspot precision of 0.88 and classification accuracy of 0.79. 3HFM has 16 mutations of which 10 are neutral and 6 mutations are observed as experimental hotspots. We predict 9 mutations to be hotspots and 7 mutations to be neutral, while 5 of the predicted hotspots are true hotspots, giving a hotspot precision of 0.56. Of the 7 predicted neutral mutations, 5 are correct, leading to an overall accuracy of 0.63 for this system. We predict a significant fraction of the hotspot mutations correctly in both of these systems, demonstrating that even when the calculated changes in binding affinity do not correlate well with experiment, the model can still be useful for practical applications.

Different optimization methods were used for calculating binding affinities: energy minimization of the wild type or mutated residue, and side-chain prediction of residues within a 0 Å or 5 Å radius of the mutation. For most of the systems studied in this work, using minimization as the MM-GBSA refinement method performs better than side-chain prediction. Although it is positive from a practical perspective that the less expensive refinement method often performs better, it is somewhat troubling that less sampling performs better. However, the reason for this may depend to some extent on the dataset studied here, which is dominated by alanine mutations. In general, mutating to a smaller residue will not produce clashes with the binding partner and therefore repacking the mutant structure is unnecessary to fit the mutated side chain. The region surrounding the mutated side chain will have less favorable van der Waals interactions than the original residue in this case, so repacking side chains may be important, but this effect does not appear to dominate the energetics for our dataset. Furthermore, allowing the structure to move, especially when not necessary, may add errors to the results. Another possible explanation for the superior minimization results is that if backbone movement is needed in order to relax a system or avoid clashes between atoms in the mutation point region, this will not be captured by the side-chain prediction method, whereas energy minimization will include both side chain and backbone atoms and will be able to relieve such strain or clashes.

While minimization alone outperforms the other refinement methods on average, it is not always the best technique for a given target or mutation. In some cases, side-chain rearrangements are necessary to fit a mutated residue and improvements in the computed binding energies can be observed with the prediction of side chains proximate to the mutation. Rearrangements allow for new intramolecular and intermolecular interactions to be accessed, which may include the formation of additional hydrogen bonds and improved packing. Optimal interactions often cannot be accessed with a minimization method due to the rough energy landscape at a protein-protein binding interface that results in getting stuck in a local minima. Side-chain prediction allows for overcoming the energy barriers and finding better interaction configurations, albeit with additional computational costs and the risk of finding incorrect results due to the additional degrees of freedom.

**Figure 3:**
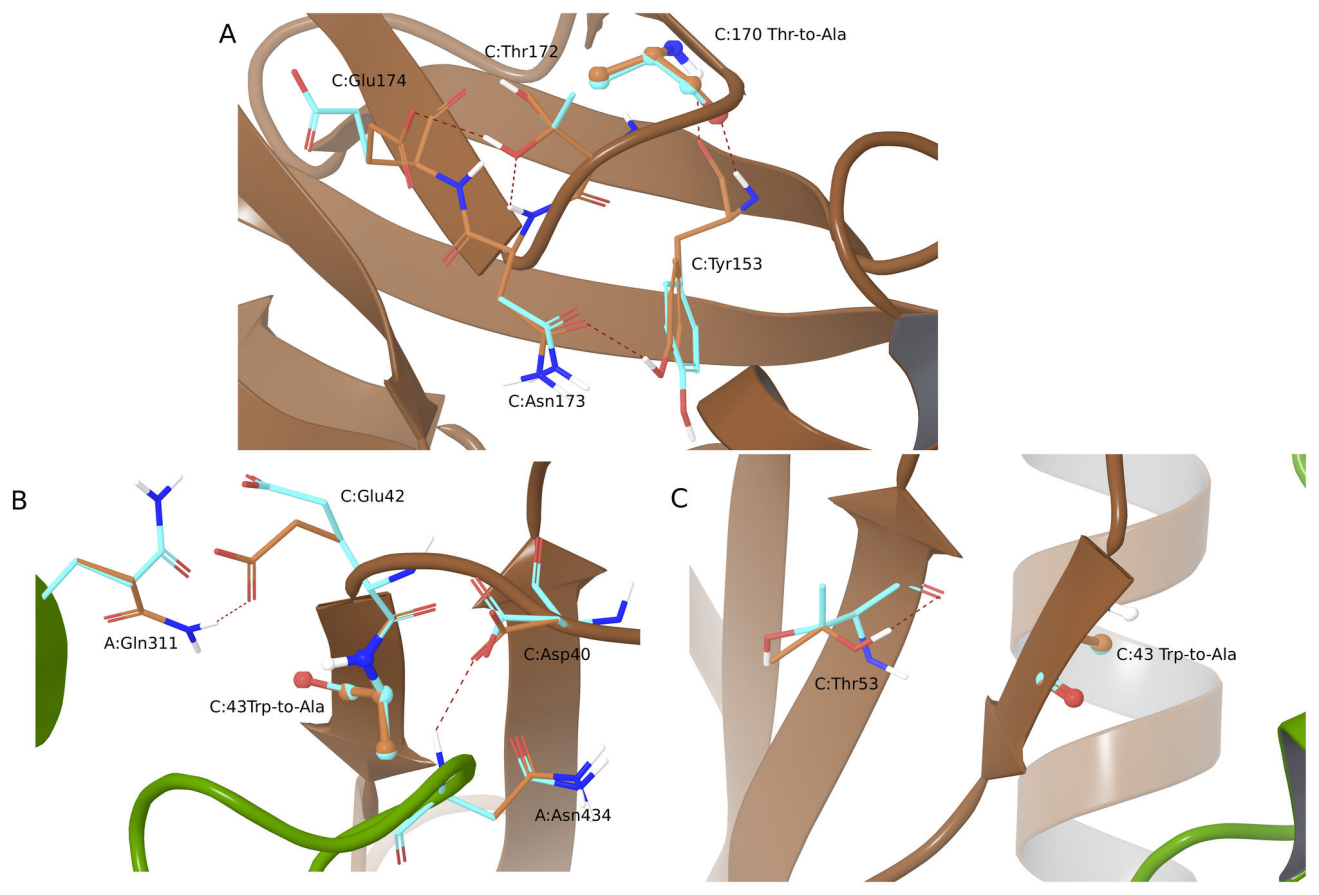
Mutations in where repacking residues nearby the mutation improves the prediction compared to minimization alone. In each case the mutant structure refined by minimization is shown in cyan and the mutant structure where a 5A radius was refined by side-chain prediction is shown in brown. The mutation residue is shown in ball-and-stick. Panel A shows the C:Thr170Ala mutation in 1AHW along with nearby residues. Panels B and C show the C:Trp43Ala mutation in 1FCC.

In two systems, repacking residues within 5 Å of the mutated residue improves results: 1AHW and 1FCC. For 1AHW, there is an increase in R^2^ value from 0.61 to 0.71 when side chains within 5 Å of the mutated residue are allowed to be searched during the calculation. We also see an increase in the hotspot precision from 0.00 to 0.67 for this system when including the side chain prediction. Figure 3 shows two examples of intra-chain and inter-chain hydrogen bond formation that are identified when side chains are flexibly sampled. Figure 3A depicts the Thr170Ala mutation in the C chain of 1AHW. The resulting structures after minimization (cyan) and side-chain prediction (brown) are superimposed and the mutation residue is represented in ball-and-stick. After side-chain prediction, we see that the side chains of Glu174 and Thr172 have rotated and are now positioned to make an internal hydrogen bond between the side-chain oxygen of Glu174 and the side-chain hydroxyl hydrogen of Thr172. Additionally, the new Thr172 side-chain conformation allows for a hydrogen bond to be formed between the side-chain oxygen of Thr172 and the backbone hydrogen of Asn173. Moreover, the side chain of Tyr153 flips by 180 degrees, enabling a hydrogen bond to form between the side-chain amide oxygen of Asn173 and the hydroxyl hydrogen of Tyr153. Side-chain prediction also results in a generally more relaxed region around the mutation point, with the total energy of the region being lowered by about 10 kcal/mol.

Another system that benefits from side-chain sampling around the mutation is 1FCC. Figures 3B and 3C show two views of the Trp43Ala mutation in the C chain. In the figure, the A chain is represented by a green ribbon, while the C chain is represented by a brown ribbon; the energy minimized system is in cyan and the side-chain predicted system in brown. For this system, the R^2^ value improves from 0.42 to 0.62 and the hotspot precision remains the same, while there is a decrease in the overall accuracy from 0.88 to 0.63. In the side-chain predicted structure, additional hydrogen bonds between the mutation chain and its binding partner are observed due to changes in the side-chain conformation. Figure 3C shows an example of two such hydrogen bonds being formed between the A and C chain of 1FCC. In the C chain, the terminal side-chain amide of Asp40 rotates so that the carbonyl oxygen atom can form a hydrogen bond with the backbone H of Asn434 in the A chain. Conformational changes in the side chains of both Glu42 (C chain) and Gln311 (A chain) facilitate a hydrogen bond between the carbonyl oxygen of Glu42 and an amide hydrogen in Gln311. Allowing the region around the mutation point to be reorganized via side-chain prediction causes the overall energy of the system to decrease by about 50 kcal/mol. Similar reorganizations are observed for other mutations in the 1AHW and 1FCC systems, contributing to the overall improvements in the model quality metrics for these systems.

**Figure 4:**
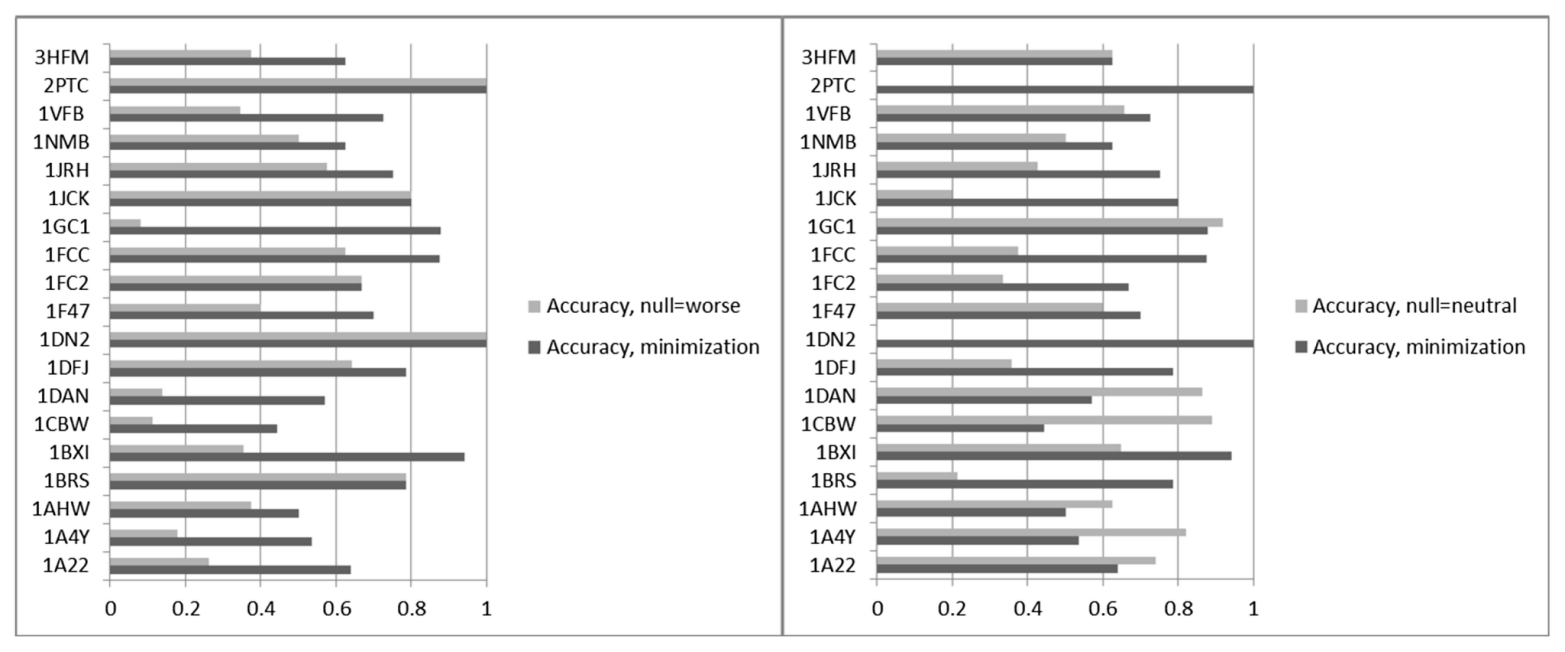
Comparison of the Accuracy metric for the minimization MM-GBSA refinement method to two different null hypotheses: the null hypothesis that all mutations are neutral, and the null hypothesis that all mutations make binding affinity worse by more than 1 kcal/mol.

Figure 4 compares the hotspot precision and accuracy classifications to a primary null hypothesis in which all mutations are predicted to be in the neutral category (the most frequent experimental outcome for this dataset) and to an alternate null hypothesis that all mutations make binding affinity worse (which is the second most frequent experimental outcome for this dataset). The alternate null hypothesis is based on outcomes from directed evolution experiments, where unfavorable single mutations combine over multiple generations to produce the desired outcome. Indeed, it has been shown that most mutational paths followed by directed evolution experiments are inaccessible because single mutations most often have a detrimental impact on the protein stability or function [33,34]. If protein-protein binding is the function of these complexes, this alternate hypothesis may be particularly relevant for these systems. For some systems (1A22, 1A4Y, 1AHW, 1CBW, 1DAN, and 1GC1), so many mutations do not change the binding affinity significantly that the null hypothesis that all mutations are neutral performs better than MM-GBSA. However, overall, our predictions of neutral and hotspot mutations are more predictive than either null hypothesis.

### Mutations that improve binding affinity

The dataset from Kortemme and Baker did not contain any single mutations that improve binding affinity by more than 1 kcal/mol, so we explored two additional targets where such mutation data has been reported. 1C4Z is a 2.6 Å resolution structure of the E6AP-Ubc47 protein-protein complex with experimental binding affinities for six single mutations (three in each binding partner), 2 of which improve binding affinity by more than 1 kcal/mol. 2OM2 is a 2.2 Å resolution structure of the GoLoco peptide bound to the a1 subunit of protein G. Similar to 1C4Z, six experimental single mutations to this complex have been published (two in protein G and four in the GoLoco peptide), 3 of which improve binding affinity by more than 1 kcal/mol [35]. Bosch et al used Rosetta to select these mutations, but since only 6 of 33 mutations predicted to improve affinity were tested, we cannot use this dataset to compare performance.

**Figure 5:**
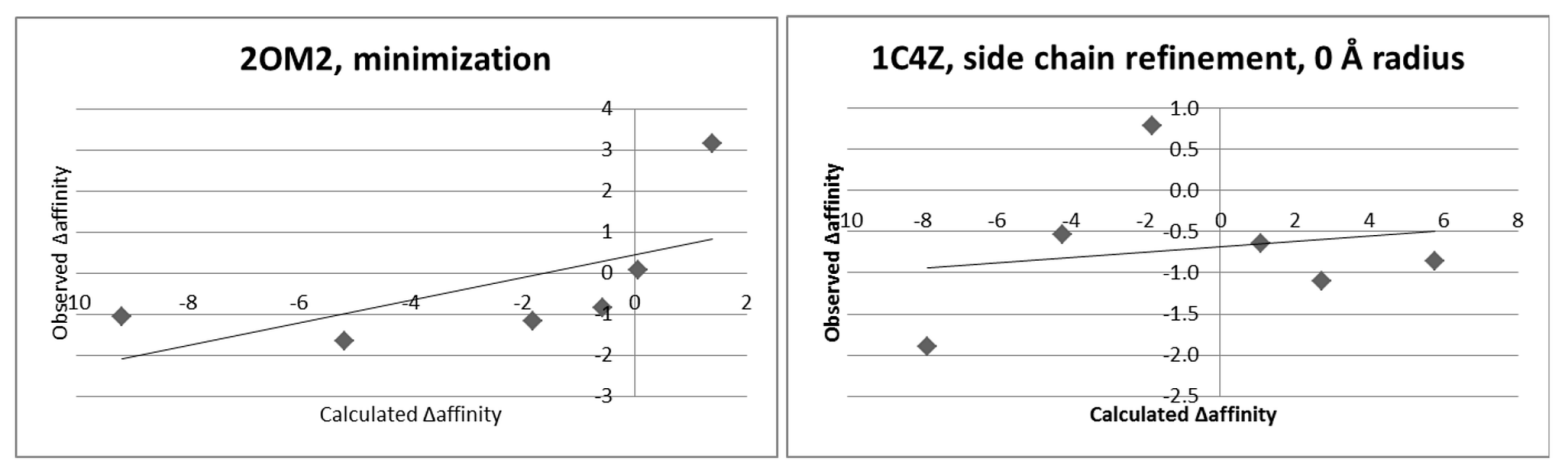
Plots of predicted versus experimental change in protein-protein binding affinity for two additional targets: 1C4Z and 2OM2. For 2OM2 the results shown are for the minimization refinement method (with only the mutated residue minimized) and for 1C4Z the results shown are for side chain refinement, with a 0 Å radius (i.e. only the mutated residue refined).

These mutations are quite different from the previously studied set in that all of them are to large residues, whereas the set from the Kortemme and Baker set was primarily to alanine. Although we do not categorize all of the 2OM2 mutations correctly using minimization as the refinement method (the accuracy and hotspot precision metrics are 0.66 and 1.0, respectively), the correlation for the 2OM2 mutations is similar to what we see for many targets in the Kortemme and Baker set (R^2^=0.38). In contrast, when we use minimization as the refinement method for 1C4Z, the hotspot predictions are all incorrect and we get a negative correlation with experiment. Similarly, when we use side-chain prediction to place the mutated residue for 1C4Z there is no correlation with experiment (R^2^=0.03) but the mutation that is predicted to most improve binding affinity is experimentally also the best mutation (see Figure 5). Comparing the mutations in these two systems highlights that the environment around each mutation position plays an important role in the quality of mutation predictions.

The 2OM2 mutations include three mutations of Gln-to-Leu, as well as one Leu-to-Tyr, one Val-to-Trp, and one Phe-to-Trp mutation. All of the mutations are at the protein-protein interface (within 3 Å of the binding partner) and all are partly solvent-exposed except for one Gln-to-Leu mutation. In all cases there is sufficient space for the mutated residue to fit. The mutations that significantly improve binding affinity are Val-to-Trp, Phe-to-Trp, and one of the Gln-to-Leu mutations. The favorable Gln-to-Leu mutation is at a partially solvent exposed position on protein G.

The 1C4Z mutations include one Gln-to-Trp mutation, two mutations from Asp (to Tyr and Trp), a Phe-to-Trp, a Lys-to-Leu, and an Ala-to-Trp. The Asp-to-Tyr in ubiquitin-protein ligase E3A and the Ala-to-Trp mutation in ubiquitin-conjugating enzyme E2 both improve binding affinity by more than 1 kcal/mol (-1.1 kcal/mol and -1.9 kcal/mol, respectively). In both cases, the minimization method predicts both of these mutations to be detrimental to binding. A:Asp641 is partly solvent-exposed in the complex, but mutation to Tyr at this position is initially placed in a clashing environment between residue D:Lys96 and A:Gln637. Minimization does not improve this and produces a highly strained and energetically unfavorable conformation of A:Tyr641. In contrast, side-chain prediction (even with only the mutated residue predicted) creates a more reasonable Tyr conformation, although it is still slightly unfavorable for binding. However, when the backbone of the mutated residue is allowed to minimize (as occurs during Prime side-chain sampling that includes the Cα-Cβ bond), the predicted delta Affinity becomes negative as the Tyr interacts more favorably with nearby D:Lys96 and A:Tyr645, and if nearby residues are included in the refinement region, the Tyr conformation becomes even more favorable and it is correctly predicted to be a hotspot. We do not know the true structure of the mutated complex (no crystal structure is available for any of the six mutants), but the refined Tyr appears to make good interactions. Similarly, initial placement of D:Trp98 is wedged tightly between A:Met653 and A:Tyr645 after mutation from the native Phe. While minimization cannot relieve this clash, side-chain refinement produces a favorable Trp conformation that is partly solvent-exposed and also makes favorable van der Waals interactions. We also predict that a neutral mutation in 1C4Z (D:Phe63-to-Trp) will improve binding affinity by making additional pockets at a buried position in the interface. It is not obvious why this is unfavorable experimentally, as the Trp appears to fit well. It is possible that water effects at the interface play a role here. Others have suggested that electrostatics can sometimes be more predictive than van der Waals for scoring mutations [36]. However, we did not explore the role of explicit water molecules in this work.

### Protonation states at protein-protein interfaces

The method of protein preparation had a significant impact on the results for this study, which is not surprising given that the importance of protein preparation for virtual screening has been shown previously [37]. By default, the Protein Preparation Wizard predicts protonation states using the PROPKA program [38,39]. PROPKA uses an empirical approach to predict pKa values by perturbing the standard pKa value for each amino acid residue with terms (whose coefficients have been fit to experimental data) that reflect local hydrogen bonding, desolvation effects, and charge-charge interactions. PROPKA has proven capable of reliably predicting pKa values (<1 log unit from experiment) for an entire protein in a matter of seconds [38]. These values are generally of comparable accuracy to those that can be obtained from computationally intensive continuum methods, and as a result the PROPKA method has found great acceptance. However, as with any empirical method, certain outlier cases can present difficulties that should be examined individually. In the studies here, there are multiple cases where the empirical protein pKa prediction program PROPKA neutralizes a buried Lys at the interface. These occur in buried hydrophobic regions, which sometimes include tryptophan residues that could make pi-cation interactions, such as Lys I:47 in 1JRH and Lys Y:96 in 3HFM. It is difficult to model the energy change for residue deprotonation upon binding because we know that protein crystal structures can change significantly when solved at different pH (for example a series of GM2AP structures have significant loop movements between PDB structure 2AG9 and 2AF9 [40]). Manually choosing the positively charged state for each of these wild-type Lys residues improves the prediction accuracy, which highlights the importance of structural inspection and manual alterations in cases where automated algorithms produce potentially incorrect states.

An energetic analysis of the above two cases provides additional insights. Default PROPKA settings result in the neutralization of Lys I:47 in 1JRH, which is a completely buried residue. We predict that mutating this neutral Lys to Ala will significantly improve binding affinity (by -7 to -8 kcal/mol) due to neutral Lys being relatively unfavorable in the crystal structure, but experimentally this mutation results in a decrease in binding affinity (by 3.5 kcal/mol). However, when the charged Lys is used for the wild type, the mutation is correctly categorized as unfavorable. Indeed, Lys can form a pi-cation interaction with Trp L:92 when it is treated in the positively charged state. Similarly, in 3HFM we predict that mutating a neutral Lys Y:96 to Ala will not significantly impact the binding affinity, but experimentally it is very unfavorable (by more than 6 kcal/mol). When the protein is prepared with the charged Lys, the predicted change in binding affinity is unfavorable because positively charged Lys is much more favorable than neutralized Lys in this position, as estimated by the MM-GBSA methodology used here. These two cases illustrate instances where the more computationally expense implicit solvent with physics-based force field outperforms the quick empirical approach for pKa prediction.

Another consideration during protein preparation is whether or not waters will be included during the preparation and during the binding energy calculations. As shown in Figures S2 and S3, when proteins with waters in the crystal structure were prepared with all waters present or with only waters that make three or more hydrogen bonds to the protein, the average correlation, accuracy, and precision results do not change significantly. For some systems the correlation is better with waters and for other systems the results are better without waters, but the differences in predictions are small and in most cases insignificant. This could either be because explicit water molecules do not play an important role in binding energy predictions for these systems or that our treatment of explicit waters was not sufficient in this work. For example, we did not allow for waters to be displaced or new waters to be introduced during the mutation process. Furthermore, a more rigorous treatment would consider the energetics of the water molecules relative to bulk solvent in addition to their presence/absence. Indeed, others have shown that explicit waters can be important in understanding molecular recognition [41] and protein binding energies for peptides [42] and small molecule systems [43]. Further work and additional datasets would be required to determine the best way to model waters when making protein mutations.

## Materials and Methods

### Datasets

The 19 datasets in Figure 1 were selected based on the work of Kortemme and Baker [13]. All protein structures were downloaded from the Protein Data Bank [44] and the mutational data was re-collected from the original references: 1A22 [45,46], 1BXI [47], 1DAN [48], 1DN2 [49], 1F47 [50], 1FCC [51], 1JRH [52,53], 1NMB [54], and 3HFM [55]. We incorporated all single mutations from the original references, including Pro-to-Ala and Gly-to-Ala mutations that were excluded in the original work. Mutation data for targets 1A4Y, 1AHW, 1BRS, 1CBW, 1DFJ, 1FC2, 1GC1, 1JCK, 1VFB, and 2PTC were collected from the Alanine Scanning Energetics database (ASEdb) [56]. This database has been updated since the Kortemme and Baker paper, so we included the currently available mutations for each target, which for most targets is a larger number than was explored by Kortemme and Baker. The two additional datasets in Figure 5 for 2OM2 and 1C4Z [57] were chosen because they include multiple mutations that improve binding affinity by more than 1 kcal/mol.

### Protein preparation

All proteins were downloaded and prepared using the Protein Preparation Wizard in the Suite2012 release, Maestro 9.3.515. The biological unit was used in all cases. For a few systems, chains far from the binding interface were deleted to reduce the size of the system while retaining everything relevant for the binding energy calculations. Several systems contained sugars, which were removed before further preparation. Defaults were used for the “preprocess” step except that missing side chains were added using Prime and waters far from het groups were not removed. PROPKA was used for the prediction of protein ionization states and ProtAssign was used for hydrogen bond optimization. After automatic hydrogen assignment, visual inspection was used to flip residues and change protonation states at the protein-protein interface when appropriate. All non-default preparation steps are described in Figure S2. All waters and SO_4_ were removed from the system before the final default restrained minimization in the Protein Preparation Wizard. A separate preparation was performed in which water molecules were retained and is presented in Figures S3 and S4.

### Residue scanning with Prime MM-GBSA

Predicted changes in binding affinity were computed using the Residue Scanning functionality in BioLuminate (version 1.0, Schrödinger, LLC, New York, NY, 2012), with command line options to specify the mutations of interest, one concurrent mutation, sequential mutations, and refinement set to either minimize or side-chain prediction. The distance for other residues to refine was set to either 0.0 (i.e. no additional residues) or 5.0 Å from the mutated residue. For systems with two binding partners in the complex, the receptor was selected as the non-mutated partner. For antibody systems, the receptor was selected as either just the antigen or both the heavy and light chains together as the receptor. The full command line invocation for minimization calculations with 0.0 Å flexible shell looked like:

*$SCHRODINGER/run $SCHRODINGER/mmshare-v21515/python/scripts/residue_scanning_backend.py -jobname <jobname> -file <.inp file of mutations> -concurrent 1 -sequential -refine_mut prime_minimize -calc e_pot,pka,sasa_polar,sasa_nonpolar,sasa_total,hydropathy,rotatable,prime_energy -dist 0.00 -receptor_asl <receptor asl> <input structure file*> 

For calculations with side-chain flexibility within 5.0 Å of the mutation the command line invocation looked like

*$SCHRODINGER/run $SCHRODINGER/mmshare-v21515/python/scripts/residue_scanning_backend.py -jobname <jobname> -file <.inp file of mutations> -concurrent 1 -sequential -refine_mut prime_minimize -calc e_pot,pka,sasa_polar,sasa_nonpolar,sasa_total,hydropathy,rotatable,prime_energy -dist 5.00 -receptor_asl <receptor asl> <input structure file*> 

**Figure 6:**
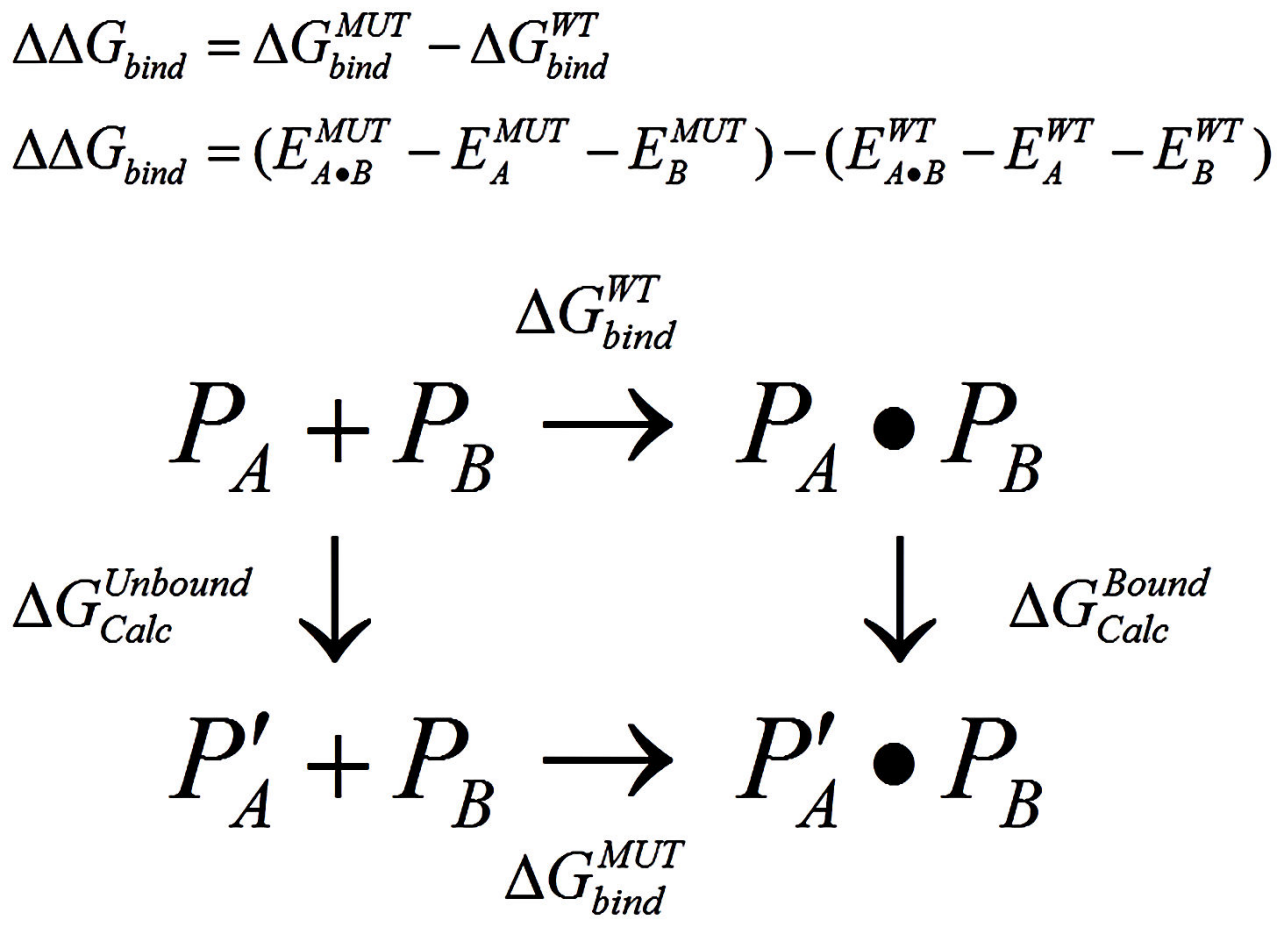
Thermodynamic cycle for calculating the net ΔΔG free energy difference between binding the wild-type protein P and the mutant protein P’. In the associated equation for ΔΔG_bind_, E is the calculated energy of each protein or complex after refinement.

Residue Scanning makes a specified list of mutations and then performs MM-GBSA refinement of the bound and unbound state for each system for both the wild type and the mutant. The predicted change in binding affinity is calculated using the equation and thermodynamic cycle in Figure 6. The state function nature of free energy allows us to calculate the net ΔΔG free energy difference by addressing the free energy changes represented by the vertical arrows in this cycle, which represent changes that are easier to simulate than the experimentally observable processes represented by the horizontal arrows.

Maestro initially places a mutated residue by setting the chi angles to those of the residue that is being replaced. Chi angles that didn’t exist in the initial residue are set to extended values (i.e. 180°). For a refinement radius greater than zero, the refinement region is defined based on the size of an arginine residue at the mutation position, and residues containing any atom within that radius of any of the arginine atoms are added to the refinement region. The refinement region is then minimized [58] or rotamers are searched for all mobile residues [59] using Prime.

A scale factor of 3 was used to relate the computed MM-GBSA energies to experimental energies. The reason for a scale factor in implicit solvent binding energy calculations has been discussed in detail elsewhere [60]. As such, a computed cutoff of 3 kcal/mol is used to predict the hotspot classes, which themselves are defined based on experimental changes in binding affinity of greater than 1.0 kcal/mol. As additional support for the scaling factor of 3, we computed the slope between prediction and experiment for systems that have at least 10 mutations and have a good correlation (R^2^ > 0.4) between the delta affinity prediction and experiment (1AHW, 1BRS, 1BXI, 1JCK, 1VFB, 1F47, 1FCC, and 1JRH). Qualified experimental values (such as >2.0) were excluded, and the remaining points were best fit by the line y = 2.91x + 0.74. While the optimal slope varies from system to system, ranging from 1.2 to 4.6, we chose the value of 3 based the average and the precedent described by Abel et al. [60] For both accuracy and hotspot precision, the minimum value is 0.0 and the maximum is 1.0.

## Supporting Information

Figure S1 **Direct comparison with Kortemme/Baker 2002 results using their metric of “fraction correct”, which assesses the fraction of experimental hotspots that are predicted to be hotspots.** The “hotspot precision” presented in the main text of our paper is the fraction of the predicted hotspots that are experimental hotspots. The “hotspot precision” was developed with a more practical question in mind: Of the mutations that are predicted to be hotspots, which of them will turn out to be experimentally validated hotspots? The MM-GBSA technique used here is the same as that used in the “Minimization” section of Figure 1 in the main paper. Cells are colored green when one method has a larger “fraction correct”.
(EPS)

Figure S2 **Protein preparation details for each system.**
(EPS)

Figure S3 **Residue scanning results with all waters included in the structure during protein preparation.**
(TIF)

Figure S4 **Residue scanning results with waters retained during protein preparation only if they make at least 3 hydrogen bonds to protein (after hydrogen bond assignment).**
(TIF)

Table S1 **Experimental and predicted mutation prediction values.**
(DOCX)

Table S2 **Residue scanning results statistics, computed separately for each residue type.**
(XLSX)
