# Supplementary figures and images for: Applying Physics-Based Scoring to Calculate Free Energies of Binding for Single Amino Acid Mutations in Protein-Protein Complexes
Source: PLoS One. 2013 Dec 10;8(12):e82849. doi: 10.1371/journal.pone.0082849 (PMC3858304; doi:10.1371/journal.pone.0082849)

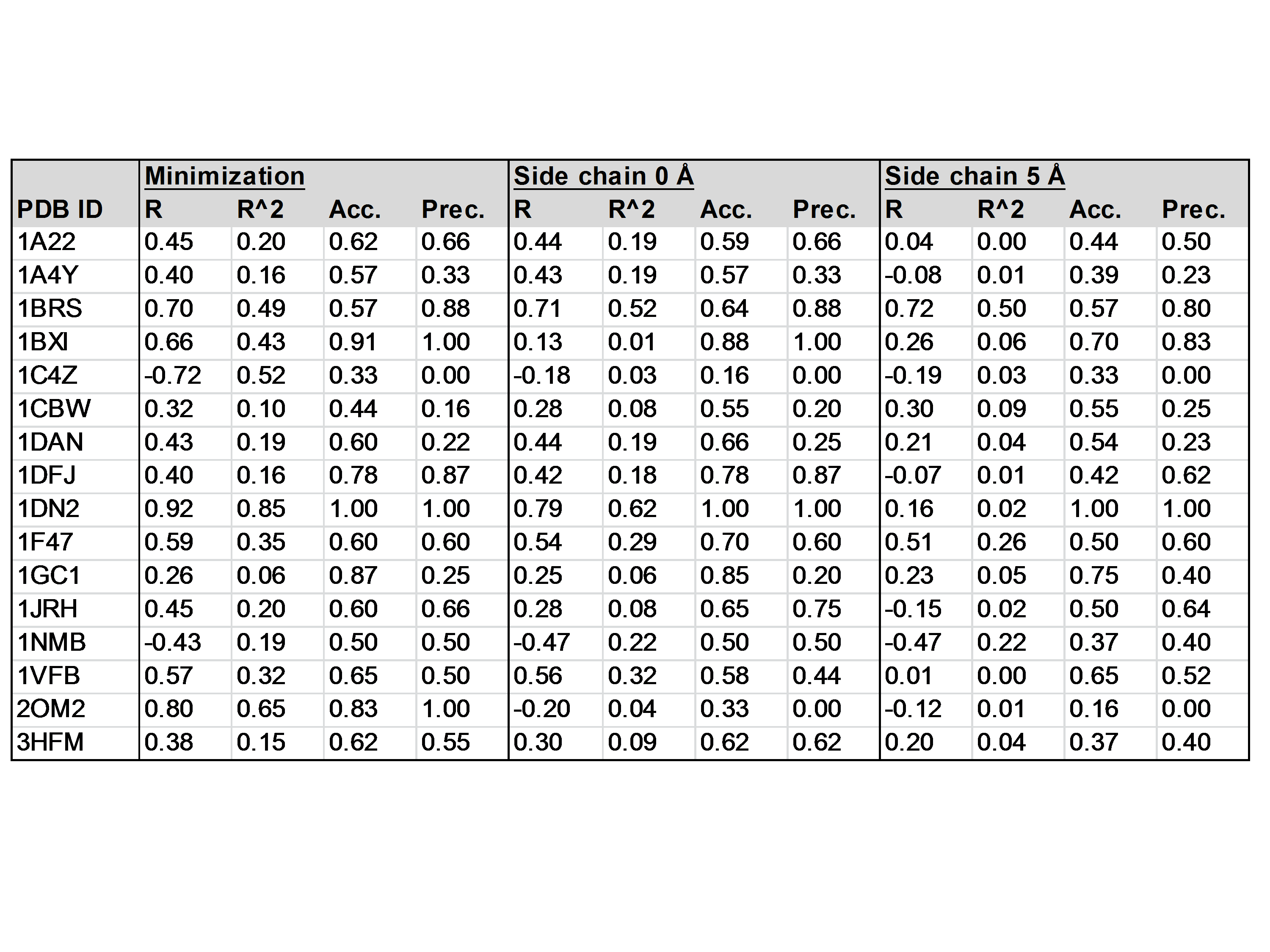

Supplement: Figure S3 — Residue scanning results with all waters included in the structure during protein preparation. (TIF) [file pone.0082849.s003.tif]

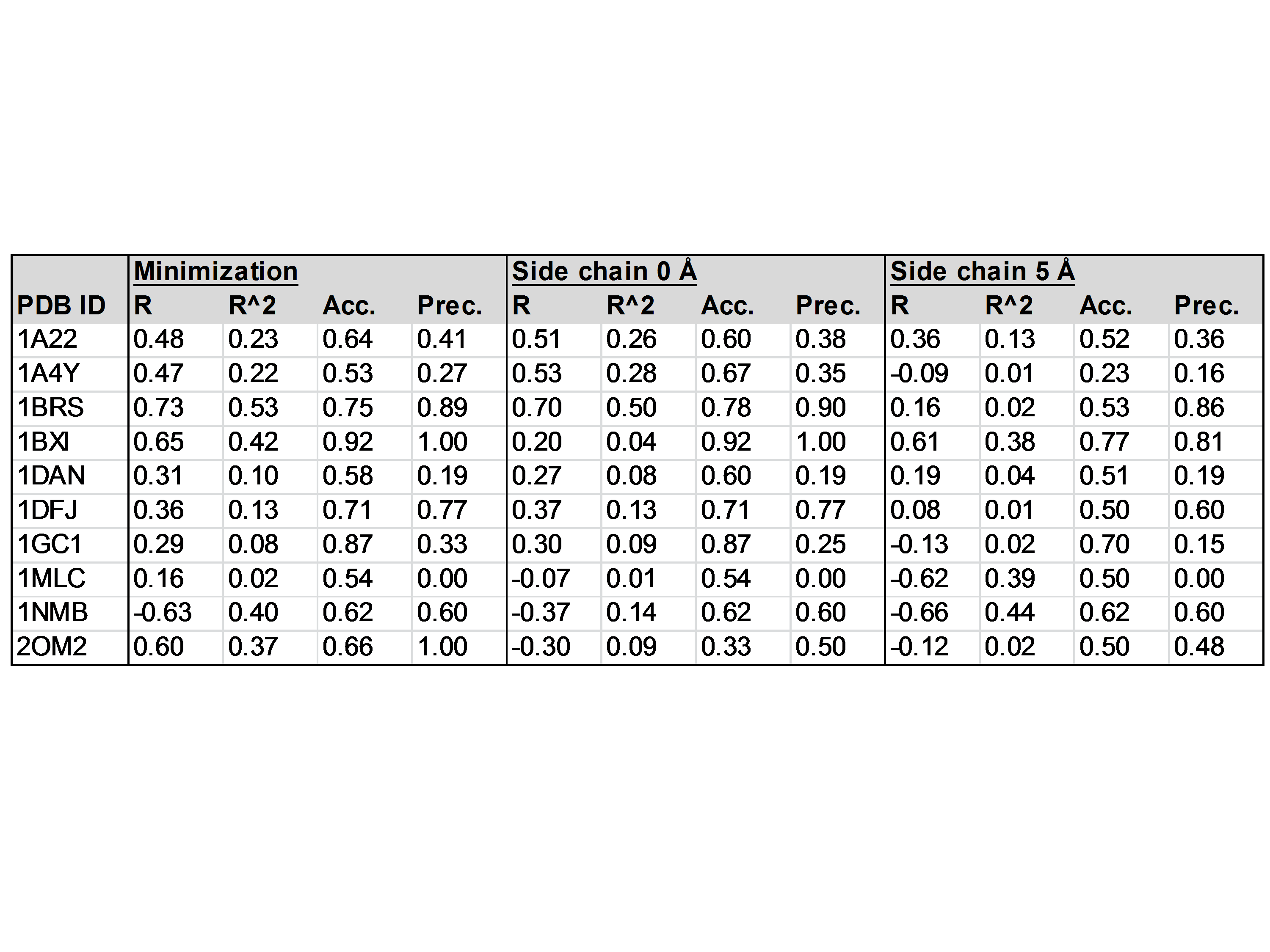

Supplement: Figure S4 — Residue scanning results with waters retained during protein preparation only if they make at least 3 hydrogen bonds to protein (after hydrogen bond assignment). (TIF) [file pone.0082849.s004.tif]
