# Supplementary material for: Applying Physics-Based Scoring to Calculate Free Energies of Binding for Single Amino Acid Mutations in Protein-Protein Complexes
Source: PLoS One. 2013 Dec 10;8(12):e82849. doi: 10.1371/journal.pone.0082849 (PMC3858304; doi:10.1371/journal.pone.0082849)
Supplement: Table S1 — Experimental and predicted mutation prediction values. (DOCX) [file pone.0082849.s005.docx]

|  |  | **Δaffinity** | **Δaffinity side chain:** | |  |
| --- | --- | --- | --- | --- | --- |
| **PDB_ID** | **Mutations** | **Minimization** | **0 Å** | **5 Å** | **Expt** |
| 1A22 | A:14(MET->ALA) | -0.70 | -0.37 | 0.53 | 0.10 |
| 1A22 | A:18(HIS->ALA) | 4.30 | 4.01 | 3.46 | -0.50 |
| 1A22 | A:21(HIS->ALA) | -1.47 | -1.40 | 4.03 | 0.20 |
| 1A22 | A:22(GLN->ALA) | 0.84 | 1.68 | 0.30 | -0.20 |
| 1A22 | A:25(PHE->ALA) | 1.34 | 1.12 | 14.91 | -0.40 |
| 1A22 | A:26(ASP->ALA) | -0.74 | -0.99 | -1.35 | -0.20 |
| 1A22 | A:29(GLN->ALA) | -0.17 | -1.25 | -0.65 | -0.60 |
| 1A22 | A:41(LYS->ALA) | 12.09 | 9.98 | -3.83 | 1.10 |
| 1A22 | A:42(TYR->ALA) | 14.30 | 14.01 | 7.76 | 0.20 |
| 1A22 | A:45(LEU->ALA) | 9.44 | 9.39 | 3.79 | 1.20 |
| 1A22 | A:46(GLN->ALA) | 10.02 | 8.11 | 1.99 | 0.10 |
| 1A22 | A:48(PRO->ALA) | 4.98 | 6.98 | 34.61 | 0.40 |
| 1A22 | A:51(SER->ALA) | 1.38 | 1.59 | 7.15 | 0.30 |
| 1A22 | A:56(GLU->ALA) | 8.02 | 7.12 | 0.14 | 0.40 |
| 1A22 | A:61(PRO->ALA) | 0.44 | 0.60 | 0.25 | 1.20 |
| 1A22 | A:62(SER->ALA) | -0.24 | -1.50 | -12.59 | 0.10 |
| 1A22 | A:63(ASN->ALA) | 4.08 | 3.78 | 1.18 | 0.30 |
| 1A22 | A:64(ARG->ALA) | 24.15 | 22.71 | 7.74 | 1.60 |
| 1A22 | A:65(GLU->ALA) | -1.28 | -2.36 | -5.72 | -0.50 |
| 1A22 | A:68(GLN->ALA) | 6.85 | 6.15 | 23.63 | 0.60 |
| 1A22 | A:164(TYR->ALA) | 1.60 | 2.80 | -39.43 | 0.30 |
| 1A22 | A:167(ARG->ALA) | 10.84 | 13.28 | -25.78 | 0.30 |
| 1A22 | A:168(LYS->ALA) | 8.11 | 7.64 | 3.51 | -0.20 |
| 1A22 | A:171(ASP->ALA) | 2.17 | 2.40 | 1.95 | 0.80 |
| 1A22 | A:172(LYS->ALA) | 3.81 | 2.91 | 4.54 | 2.00 |
| 1A22 | A:174(GLU->ALA) | 1.74 | 2.03 | 3.03 | -0.90 |
| 1A22 | A:175(THR->ALA) | 9.18 | 9.20 | 9.87 | 2.00 |
| 1A22 | A:176(PHE->ALA) | 2.14 | 2.03 | 2.33 | 1.90 |
| 1A22 | A:178(ARG->ALA) | 8.19 | 8.57 | 45.20 | 2.40 |
| 1A22 | A:179(ILE->ALA) | 2.95 | 4.06 | 19.62 | 0.80 |
| 1A22 | A:183(ARG->ALA) | 0.26 | 0.47 | -5.16 | 0.50 |
| 1A22 | A:186(GLU->ALA) | -0.43 | -0.31 | -0.32 | 0.00 |
| 1A22 | B:242(GLU->ALA) | -0.69 | -0.45 | -3.33 | 0.18 |
| 1A22 | B:243(ARG->ALA) | 16.91 | 18.92 | 21.83 | 2.12 |
| 1A22 | B:244(GLU->ALA) | 3.46 | 2.63 | 2.65 | 1.69 |
| 1A22 | B:270(ARG->ALA) | 2.41 | 2.43 | 15.12 | 0.69 |
| 1A22 | B:271(ARG->ALA) | 20.54 | 17.47 | 11.55 | 0.54 |
| 1A22 | B:272(ASN->ALA) | -0.28 | -0.29 | 26.46 | 0.28 |
| 1A22 | B:273(THR->ALA) | -0.01 | 0.12 | 0.04 | 0.11 |
| 1A22 | B:274(GLN->ALA) | 0.50 | -0.36 | -0.33 | 0.00 |
| 1A22 | B:275(GLU->ALA) | -1.48 | -3.92 | 0.02 | -0.10 |
| 1A22 | B:276(TRP->ALA) | 14.45 | 14.44 | 38.97 | 0.51 |
| 1A22 | B:277(THR->ALA) | -0.02 | 0.03 | 0.66 | 0.20 |
| 1A22 | B:280(TRP->ALA) | 0.11 | 0.00 | 8.56 | -0.02 |
| 1A22 | B:298(SER->ALA) | 0.48 | 0.53 | 5.02 | -0.05 |
| 1A22 | B:301(THR->ALA) | 0.37 | 0.38 | -3.31 | 1.76 |
| 1A22 | B:302(SER->ALA) | 7.42 | 0.55 | 2.65 | -0.20 |
| 1A22 | B:303(ILE->ALA) | 1.52 | 1.71 | 6.61 | 1.61 |
| 1A22 | B:304(TRP->ALA) | 19.60 | 19.66 | 25.21 | 4.50 |
| 1A22 | B:305(ILE->ALA) | 2.09 | 1.97 | 10.70 | 1.94 |
| 1A22 | B:306(PRO->ALA) | 3.97 | 4.15 | 93.17 | 3.31 |
| 1A22 | B:320(GLU->ALA) | 2.81 | 2.37 | 33.08 | -0.19 |
| 1A22 | B:321(LYS->ALA) | 0.08 | 0.25 | -2.20 | 0.08 |
| 1A22 | B:324(SER->ALA) | 1.48 | 3.80 | 0.84 | 0.28 |
| 1A22 | B:326(ASP->ALA) | -4.84 | -5.25 | 2.18 | 0.99 |
| 1A22 | B:327(GLU->ALA) | 12.79 | 13.13 | 6.09 | 0.97 |
| 1A22 | B:364(ASP->ALA) | 10.67 | 10.64 | -8.93 | 1.49 |
| 1A22 | B:365(ILE->ALA) | 0.67 | 0.51 | 2.46 | 2.13 |
| 1A22 | B:366(GLN->ALA) | -0.32 | 1.03 | -3.24 | 0.02 |
| 1A22 | B:367(LYS->ALA) | -3.11 | -3.75 | -2.16 | -0.02 |
| 1A22 | B:369(TRP->ALA) | 21.68 | 21.13 | 18.86 | 4.50 |
| 1A22 | B:371(VAL->ALA) | 3.49 | 3.51 | 2.74 | -0.64 |
| 1A22 | B:394(THR->ALA) | -0.02 | -0.01 | -0.06 | 0.20 |
| 1A22 | B:395(THR->ALA) | -0.12 | -0.19 | -0.34 | -0.09 |
| 1A22 | B:415(LYS->ALA) | 1.06 | 0.78 | -17.90 | 0.79 |
| 1A22 | B:416(GLN->ALA) | 0.28 | 0.08 | -2.40 | 0.89 |
| 1A22 | B:417(ARG->ALA) | 4.18 | 4.47 | 9.63 | 0.28 |
| 1A22 | B:418(ASN->ALA) | 9.81 | 10.92 | 3.17 | 0.30 |
| 1A22 | B:419(SER->ALA) | 7.19 | -1.10 | -3.57 | 0.03 |
|  |  |  |  |  |  |
| 1A4Y | A:261(TRP->ALA) | 5.85 | 7.78 | 5.04 | 0.10 |
| 1A4Y | A:263(TRP->ALA) | 8.79 | 8.54 | 11.91 | 1.20 |
| 1A4Y | A:287(GLU->ALA) | 0.51 | 0.22 | 19.59 | 0.10 |
| 1A4Y | A:289(SER->ALA) | 3.29 | 2.73 | 2.55 | 0.00 |
| 1A4Y | A:318(TRP->ALA) | 14.88 | 14.58 | 9.50 | 1.50 |
| 1A4Y | A:320(LYS->ALA) | 3.95 | 6.24 | 5.12 | -0.30 |
| 1A4Y | A:344(GLU->ALA) | -0.66 | 0.34 | -9.27 | 0.20 |
| 1A4Y | A:375(TRP->ALA) | 12.30 | 11.81 | 32.51 | 1.00 |
| 1A4Y | A:401(GLU->ALA) | -2.34 | -2.36 | -5.16 | 0.90 |
| 1A4Y | A:457(ARG->ALA) | -0.60 | -0.69 | 0.21 | -0.20 |
| 1A4Y | A:459(ILE->ALA) | 3.36 | 2.89 | 5.12 | 0.70 |
| 1A4Y | A:434(TYR->ALA) | 13.49 | 12.23 | -15.52 | 3.30 |
| 1A4Y | A:435(ASP->ALA) | 5.28 | 5.43 | -1.95 | 3.50 |
| 1A4Y | A:437(TYR->ALA) | 13.58 | 16.48 | 12.89 | 0.80 |
| 1A4Y | B:5(ARG->ALA) | 23.96 | 23.93 | 38.37 | 2.30 |
| 1A4Y | B:8(HIS->ALA) | -0.39 | -1.22 | -49.49 | 0.90 |
| 1A4Y | B:12(GLN->ALA) | -2.77 | -1.05 | 39.47 | 0.30 |
| 1A4Y | B:13(HIS->ALA) | -2.09 | -2.47 | 9.90 | -0.30 |
| 1A4Y | B:31(ARG->ALA) | 23.96 | 8.59 | 6.14 | 0.20 |
| 1A4Y | B:32(ARG->ALA) | 10.60 | 17.10 | 18.38 | 0.90 |
| 1A4Y | B:33(ARG->ALA) | 4.17 | 3.83 | 2.78 | 0.30 |
| 1A4Y | B:66(ARG->ALA) | 3.04 | 2.87 | 3.39 | 0.20 |
| 1A4Y | B:68(ASN->ALA) | 3.70 | 0.98 | -71.51 | 0.20 |
| 1A4Y | B:70(ARG->ALA) | 1.67 | 1.68 | 3.73 | -0.20 |
| 1A4Y | B:84(HIE->ALA) | 2.66 | 2.85 | 1.39 | 0.20 |
| 1A4Y | B:89(TRP->ALA) | 3.57 | 3.84 | 5.35 | 0.20 |
| 1A4Y | B:108(GLU->ALA) | 1.97 | 2.19 | 14.95 | -0.30 |
| 1A4Y | B:114(HIS->ALA) | 3.75 | 3.67 | -92.26 | 0.65 |
|  |  |  |  |  |  |
| 1AHW | C:156(TYR->ALA) | 2.37 | 2.19 | 154.95 | 4.00 |
| 1AHW | C:167(THR->ALA) | -0.81 | -1.10 | 19.73 | 0.00 |
| 1AHW | C:170(THR->ALA) | 0.59 | 1.19 | -8.25 | 1.00 |
| 1AHW | C:176(LEU->ALA) | -0.32 | -0.04 | -3.03 | 1.00 |
| 1AHW | C:178(ASP->ALA) | -3.15 | -2.91 | -2.32 | -0.50 |
| 1AHW | C:197(THR->ALA) | 1.04 | 0.99 | 0.89 | 1.30 |
| 1AHW | C:198(VAL->ALA) | 0.42 | 0.35 | 0.37 | -0.30 |
| 1AHW | C:199(ASN->ALA) | -0.22 | 0.29 | 3.00 | 1.10 |
|  |  |  |  |  |  |
| 1BRS | A:27(LYS->ALA) | 13.59 | 11.91 | 7.09 | 5.40 |
| 1BRS | A:54(ASP->ALA) | -2.26 | -2.18 | -8.82 | -0.80 |
| 1BRS | A:58(ASN->ALA) | 0.08 | 0.25 | -3.77 | 3.10 |
| 1BRS | A:59(ARG->ALA) | 28.98 | 29.90 | 21.65 | 5.20 |
| 1BRS | A:60(GLU->ALA) | 3.65 | 6.64 | 32.90 | -0.20 |
| 1BRS | A:73(GLU->ALA) | -5.90 | -5.71 | -0.10 | 2.80 |
| 1BRS | A:87(ARG->ALA) | 16.74 | 16.84 | 11.98 | 5.50 |
| 1BRS | A:102(HIE->ALA) | 23.00 | 20.47 | -8.30 | 6.00 |
| 1BRS | D:29(TYR->ALA) | 16.73 | 16.04 | 16.30 | 3.40 |
| 1BRS | D:35(ASP->ALA) | 8.23 | 8.08 | 14.06 | 4.50 |
| 1BRS | D:39(ASP->ALA) | 18.82 | 17.68 | 17.70 | 7.70 |
| 1BRS | D:42(THR->ALA) | 3.66 | 3.82 | 1.97 | 1.80 |
| 1BRS | D:76(GLU->ALA) | 10.69 | 6.62 | 4.33 | 1.30 |
| 1BRS | D:80(GLU->ALA) | -0.65 | -0.02 | -0.12 | 0.50 |
|  |  |  |  |  |  |
| 1BXI | A:23(CYS->ALA) | 0.08 | 1.60 | -8.96 | 0.92 |
| 1BXI | A:24(ASN->ALA) | -0.50 | 0.11 | -2.64 | 0.14 |
| 1BXI | A:26(ASP->ALA) | 0.85 | 0.78 | 1.77 | 0.34 |
| 1BXI | A:27(THR->ALA) | 2.05 | 1.36 | -1.16 | 0.73 |
| 1BXI | A:28(SER->ALA) | -0.20 | -0.06 | -1.11 | 0.17 |
| 1BXI | A:29(SER->ALA) | -0.75 | -0.12 | 0.17 | 0.96 |
| 1BXI | A:30(GLU->ALA) | 19.33 | 18.03 | 7.65 | 1.41 |
| 1BXI | A:31(GLU->ALA) | 0.34 | 0.42 | -8.08 | 0.31 |
| 1BXI | A:32(GLU->ALA) | 2.08 | 1.75 | 0.87 | 0.22 |
| 1BXI | A:33(LEU->ALA) | 6.60 | 6.55 | 8.83 | 3.42 |
| 1BXI | A:34(VAL->ALA) | 4.94 | 4.72 | 8.77 | 2.58 |
| 1BXI | A:35(LYS->ALA) | -1.58 | -1.54 | -1.39 | 0.19 |
| 1BXI | A:36(LEU->ALA) | -0.11 | -0.11 | -0.04 | 0.91 |
| 1BXI | A:37(VAL->ALA) | 3.11 | 3.27 | 2.91 | 1.66 |
| 1BXI | A:38(THR->ALA) | 2.51 | 2.56 | 24.16 | 0.90 |
| 1BXI | A:41(GLU->ALA) | 10.81 | 10.26 | 12.49 | 2.08 |
| 1BXI | A:42(GLU->ALA) | 1.29 | 1.21 | 1.47 | 0.66 |
| 1BXI | A:44(THR->ALA) | 0.02 | 0.05 | 0.35 | 0.30 |
| 1BXI | A:45(GLU->ALA) | 1.17 | 1.18 | 1.06 | 0.21 |
| 1BXI | A:46(HIE->ALA) | -0.17 | -0.18 | -0.23 | 0.83 |
| 1BXI | A:47(PRO->ALA) | -0.05 | 0.09 | 10.24 | 0.44 |
| 1BXI | A:48(SER->ALA) | -0.01 | 0.05 | -0.19 | 0.01 |
| 1BXI | A:49(GLY->ALA) | 4.27 | 50.75 | -0.91 | 1.49 |
| 1BXI | A:50(SER->ALA) | 3.89 | 2.74 | 8.71 | 2.19 |
| 1BXI | A:51(ASP->ALA) | 5.14 | 5.02 | 4.42 | 5.92 |
| 1BXI | A:52(LEU->ALA) | -0.07 | -0.15 | 1.36 | 0.60 |
| 1BXI | A:53(ILE->ALA) | 1.12 | 1.90 | 1.40 | 0.85 |
| 1BXI | A:54(TYR->ALA) | 18.43 | 16.60 | 20.39 | 4.83 |
| 1BXI | A:55(TYR->ALA) | 12.77 | 12.68 | 18.59 | 4.63 |
| 1BXI | A:56(PRO->ALA) | 2.56 | 2.51 | 2.39 | 1.24 |
| 1BXI | A:60(ASP->ALA) | 0.90 | 0.92 | 1.00 | 0.51 |
| 1BXI | A:63(SER->ALA) | 0.33 | 0.10 | 1.59 | 0.87 |
| 1BXI | A:68(VAL->ALA) | -0.12 | -0.05 | -0.01 | 1.86 |
| 1BXI | A:69(ASN->ALA) | 0.07 | -0.01 | -0.13 | 0.28 |
|  |  |  |  |  |  |
| 1CBW | D:11(THR->ALA) | 3.51 | 1.71 | 1.54 | 0.20 |
| 1CBW | D:13(PRO->ALA) | 3.62 | 3.87 | 3.84 | -0.10 |
| 1CBW | D:15(LYS->ALA) | 4.05 | 1.35 | 2.78 | 2.00 |
| 1CBW | D:17(ARG->ALA) | 19.73 | 19.40 | 19.94 | 0.50 |
| 1CBW | D:19(ILE->ALA) | 3.66 | 2.86 | 2.94 | 0.10 |
| 1CBW | D:20(ARG->ALA) | 0.55 | 0.58 | 0.24 | 0.30 |
| 1CBW | D:34(VAL->ALA) | 2.92 | 2.91 | 5.70 | 0.00 |
| 1CBW | D:39(ARG->ALA) | 7.70 | 8.44 | 9.31 | 0.20 |
| 1CBW | D:46(LYS->ALA) | -0.86 | -0.87 | -0.99 | 0.10 |
|  |  |  |  |  |  |
| 1DAN | U:208(GLU->ALA) | 6.48 | 6.18 | 6.46 | 0.00 |
| 1DAN | U:207(VAL->ALA) | 8.84 | 8.76 | 15.40 | -0.20 |
| 1DAN | U:203(THR->ALA) | 0.57 | 1.53 | 4.69 | 0.15 |
| 1DAN | U:195(SER->ALA) | -0.16 | -0.17 | -0.34 | 0.00 |
| 1DAN | U:185(TYR->ALA) | 0.10 | 0.14 | -0.15 | -0.30 |
| 1DAN | U:181(LYS->ALA) | -3.02 | -2.86 | -3.09 | 0.01 |
| 1DAN | U:176(LEU->ALA) | -0.01 | 0.00 | -0.07 | 0.02 |
| 1DAN | U:172(THR->ALA) | -0.05 | 0.01 | -0.02 | -0.01 |
| 1DAN | U:169(LYS->ALA) | -0.75 | -0.79 | -0.80 | 0.10 |
| 1DAN | U:167(THR->ALA) | 0.00 | -0.03 | -0.09 | 0.20 |
| 1DAN | U:164(GLY->ARG) | 1.93 | 0.91 | 1.67 | -0.20 |
| 1DAN | U:163(SER->ALA) | -0.62 | 2.61 | -46.45 | 0.02 |
| 1DAN | U:158(TRP->PHE) | 2.88 | 1.37 | 19.63 | 0.15 |
| 1DAN | U:156(TYR->LEU) | 0.03 | -0.66 | -3.65 | 0.15 |
| 1DAN | U:152(ILE->ALA) | -0.09 | -0.11 | -0.48 | 0.20 |
| 1DAN | U:145(ASP->ALA) | 0.30 | 0.23 | 0.68 | 0.00 |
| 1DAN | U:144(ARG->ALA) | -0.57 | -1.01 | 0.45 | 0.00 |
| 1DAN | U:140(PHE->ALA) | 8.95 | 9.03 | 13.03 | 1.30 |
| 1DAN | U:139(THR->ALA) | -0.11 | -0.01 | 0.07 | 0.00 |
| 1DAN | U:135(ARG->ALA) | 9.94 | 10.60 | 13.08 | 0.50 |
| 1DAN | U:133(LEU->ALA) | 16.50 | 14.75 | 15.57 | -0.01 |
| 1DAN | U:129(ASP->ALA) | 0.90 | 0.88 | 7.36 | -0.01 |
| 1DAN | U:128(GLU->ALA) | 5.22 | 4.70 | 2.86 | 0.10 |
| 1DAN | U:122(LYS->ALA) | -1.08 | -1.16 | -1.12 | -0.15 |
| 1DAN | U:99(GLU->ALA) | 7.05 | 3.17 | 2.80 | -0.15 |
| 1DAN | U:94(TYR->ALA) | 26.41 | 25.00 | -9.35 | 1.10 |
| 1DAN | T:68(LYS->ALA) | 0.61 | -0.08 | -6.62 | -0.10 |
| 1DAN | T:58(ASP->GLU) | 0.43 | 0.44 | 0.75 | 1.40 |
| 1DAN | T:58(ASP->ALA) | 1.52 | 2.10 | -0.22 | 2.00 |
| 1DAN | T:52(THR->ALA) | -0.38 | -0.37 | -0.28 | 0.40 |
| 1DAN | T:50(PHE->ALA) | 13.45 | 13.52 | 8.52 | 0.50 |
| 1DAN | T:48(LYS->ALA) | 8.52 | 8.60 | -11.08 | 0.40 |
| 1DAN | T:47(SER->ALA) | 0.96 | 1.87 | 1.95 | 0.05 |
| 1DAN | T:46(LYS->ALA) | 3.35 | 2.85 | 5.19 | 0.20 |
| 1DAN | T:45(TRP->PHE) | 2.17 | -0.35 | 4.34 | 1.30 |
| 1DAN | T:44(ASP->ALA) | 2.48 | 2.58 | 2.13 | 0.80 |
| 1DAN | T:42(SER->ALA) | -0.68 | -0.56 | -0.87 | -0.05 |
| 1DAN | T:41(LYS->ALA) | 5.41 | 4.07 | 3.79 | 0.30 |
| 1DAN | T:37(GLN->ALA) | 4.27 | 4.04 | 28.20 | 0.60 |
| 1DAN | T:28(LYS->ALA) | 0.55 | 0.60 | 0.54 | 0.15 |
| 1DAN | T:26(GLU->ALA) | -0.44 | -0.46 | -0.67 | 0.10 |
| 1DAN | T:25(TRP->PHE) | -0.06 | -0.08 | 0.22 | 0.60 |
| 1DAN | T:24(GLU->ALA) | 4.60 | 4.47 | 12.56 | 0.70 |
| 1DAN | T:22(ILE->ALA) | 6.36 | 6.78 | 4.71 | 0.70 |
| 1DAN | T:21(THR->ALA) | 3.73 | 3.24 | -7.48 | -0.20 |
| 1DAN | T:20(LYS->ARG) | 0.86 | -0.47 | -2.73 | 1.70 |
| 1DAN | T:20(LYS->ALA) | 8.93 | 9.08 | 8.95 | 2.60 |
| 1DAN | T:18(ASN->ALA) | 10.55 | 13.61 | 9.66 | 0.20 |
| 1DAN | T:17(THR->ALA) | 3.45 | 3.45 | 5.14 | 0.10 |
| 1DAN | T:15(LYS->ALA) | 0.27 | 0.02 | -21.87 | -0.40 |
| 1DAN | T:14(TRP->PHE) | 9.89 | 8.49 | 80.02 | 0.70 |
|  |  |  |  |  |  |
| 1DFJ | I:202(GLU->ALA) | 2.96 | 6.32 | -59.49 | 1.00 |
| 1DFJ | I:257(TRP->ALA) | 5.77 | -2.60 | -54.47 | 1.30 |
| 1DFJ | I:259(TRP->ALA) | 12.32 | 11.75 | 50.51 | 2.20 |
| 1DFJ | I:283(GLU->ALA) | 4.08 | 4.32 | -26.19 | 1.30 |
| 1DFJ | I:285(SER->ALA) | 0.48 | 0.59 | 17.55 | 0.80 |
| 1DFJ | I:314(TRP->ALA) | 2.93 | 2.99 | 14.76 | 1.00 |
| 1DFJ | I:316(LYS->ALA) | 3.10 | 4.37 | 8.63 | 1.30 |
| 1DFJ | I:340(GLU->ALA) | 1.53 | 9.11 | 5.18 | 1.60 |
| 1DFJ | I:397(GLU->ALA) | 4.38 | 4.28 | 4.43 | 1.30 |
| 1DFJ | I:453(ARG->ALA) | 6.52 | -0.51 | 5.11 | 0.80 |
| 1DFJ | I:455(ILE->ALA) | 2.34 | 2.79 | 20.11 | 0.30 |
| 1DFJ | I:430(TYR->ALA) | 11.82 | 11.82 | 13.20 | 5.90 |
| 1DFJ | I:431(ASP->ALA) | 2.30 | 1.52 | 1.83 | 3.60 |
| 1DFJ | I:433(TYR->ALA) | 20.82 | 21.08 | 21.13 | 2.60 |
|  |  |  |  |  |  |
| 1DN2 | A:434(ASN->ALA) | 4.13 | 6.92 | 7.41 | 1.50 |
| 1DN2 | A:435(HIS->ALA) | 6.51 | 6.05 | 5.79 | 1.50 |
| 1DN2 | A:436(TYR->ALA) | 8.15 | 12.59 | 30.41 | 1.50 |
| 1DN2 | E:10(VAL->ALA) | 13.39 | 13.59 | 30.35 | 2.00 |
| 1DN2 | E:11(TRP->ALA) | 18.00 | 22.27 | 8.75 | 2.00 |
|  |  |  |  |  |  |
| 1F47 | A:4(ASP->ALA) | 0.64 | -0.28 | 0.07 | 0.69 |
| 1F47 | A:5(TYR->ALA) | 16.32 | 16.06 | 8.12 | 0.87 |
| 1F47 | A:6(LEU->ALA) | 8.79 | 9.10 | 11.19 | 0.92 |
| 1F47 | A:7(ASP->ALA) | 2.63 | 2.18 | 1.33 | 1.73 |
| 1F47 | A:8(ILE->ALA) | 14.92 | 15.00 | 14.68 | 2.51 |
| 1F47 | A:9(PRO->ALA) | 1.09 | 1.18 | 0.71 | -0.06 |
| 1F47 | A:11(PHE->ALA) | 13.04 | 13.98 | 9.03 | 2.44 |
| 1F47 | A:12(LEU->ALA) | 7.41 | 7.37 | 7.93 | 2.29 |
| 1F47 | A:14(LYS->ALA) | -0.33 | -0.10 | 0.68 | -0.04 |
| 1F47 | A:15(GLN->ALA) | 0.37 | 0.29 | 1.31 | -0.05 |
|  |  |  |  |  |  |
| 1FC2 | C:147(ASN->ALA) | -2.50 | 1.63 | -6.42 | 0.60 |
| 1FC2 | C:150(ILE->ALA) | 4.68 | 5.01 | -4.08 | 2.20 |
| 1FC2 | C:154(LYS->ALA) | 1.47 | 1.24 | 2.00 | 1.20 |
|  |  |  |  |  |  |
| 1FCC | C:25(THR->ALA) | 0.49 | 0.36 | 1.85 | 0.24 |
| 1FCC | C:27(GLU->ALA) | 16.33 | 16.31 | 19.27 | 4.90 |
| 1FCC | C:28(LYS->ALA) | 16.67 | 13.68 | -3.67 | 1.30 |
| 1FCC | C:31(LYS->ALA) | 6.32 | 7.39 | 3.92 | 3.50 |
| 1FCC | C:35(ASN->ALA) | 12.24 | 9.14 | 2.53 | 2.40 |
| 1FCC | C:40(ASP->ALA) | 1.05 | 2.21 | 0.36 | 0.30 |
| 1FCC | C:42(GLU->ALA) | -4.47 | -4.17 | -3.43 | 0.40 |
| 1FCC | C:43(TRP->ALA) | 9.29 | 8.52 | 4.09 | 3.80 |
|  |  |  |  |  |  |
| 1GC1 | C:1(LYS->ALA) | -0.07 | -0.33 | -0.06 | 0.06 |
| 1GC1 | C:2(LYS->ALA) | -0.66 | -0.39 | -0.49 | -0.02 |
| 1GC1 | C:8(LYS->ALA) | 0.00 | -0.14 | -0.03 | 0.10 |
| 1GC1 | C:10(ASP->ALA) | 0.26 | 0.27 | 0.26 | 0.00 |
| 1GC1 | C:11(THR->ALA) | -0.01 | -0.03 | 0.06 | 0.00 |
| 1GC1 | C:15(THR->ALA) | -0.03 | 0.03 | -0.06 | 0.32 |
| 1GC1 | C:19(SER->ALA) | 0.03 | 0.03 | -0.43 | 0.00 |
| 1GC1 | C:20(GLN->ALA) | -0.05 | -0.08 | -0.05 | -0.02 |
| 1GC1 | C:21(LYS->ALA) | -0.21 | -0.34 | -0.47 | -0.13 |
| 1GC1 | C:22(LYS->ALA) | -0.39 | -0.24 | -1.57 | 0.24 |
| 1GC1 | C:23(SER->ALA) | -0.42 | 0.03 | -2.13 | 0.29 |
| 1GC1 | C:25(GLN->ALA) | 1.59 | 0.63 | 3.87 | 0.03 |
| 1GC1 | C:27(HIS->ALA) | -1.81 | -0.84 | 1.58 | 0.28 |
| 1GC1 | C:29(LYS->ALA) | 11.75 | 11.81 | 7.80 | 0.59 |
| 1GC1 | C:30(ASN->ALA) | -0.11 | -0.12 | 4.93 | 0.17 |
| 1GC1 | C:31(SER->ALA) | 0.03 | 0.01 | -0.05 | 0.10 |
| 1GC1 | C:32(ASN->ALA) | 0.05 | -2.82 | -1.94 | 0.18 |
| 1GC1 | C:33(GLN->ALA) | 0.45 | -0.42 | -3.31 | 0.10 |
| 1GC1 | C:35(LYS->ALA) | -2.62 | 1.99 | 7.23 | 0.32 |
| 1GC1 | C:39(ASN->ALA) | -0.36 | -0.43 | -1.35 | 0.46 |
| 1GC1 | C:40(GLN->ALA) | 4.51 | 3.37 | 2.20 | -0.41 |
| 1GC1 | C:42(SER->ALA) | 2.02 | -1.08 | 49.96 | 0.00 |
| 1GC1 | C:44(LEU->ALA) | 1.09 | 2.12 | 73.23 | 1.04 |
| 1GC1 | C:45(THR->ALA) | 1.97 | 1.34 | -4.93 | -0.15 |
| 1GC1 | C:49(SER->ALA) | -0.11 | -0.10 | 0.07 | 0.60 |
| 1GC1 | C:50(LYS->ALA) | -0.21 | -0.06 | -0.08 | 0.05 |
| 1GC1 | C:52(ASN->ALA) | 0.40 | 0.57 | -0.24 | 0.70 |
| 1GC1 | C:53(ASP->ALA) | 0.48 | 0.49 | 0.55 | 0.30 |
| 1GC1 | C:56(ASP->ALA) | 0.38 | 0.33 | 5.29 | -0.07 |
| 1GC1 | C:58(ARG->ALA) | -0.42 | -0.30 | 0.17 | 0.13 |
| 1GC1 | C:59(ARG->ALA) | 15.76 | 8.17 | -148.06 | 1.16 |
| 1GC1 | C:60(SER->ALA) | 0.01 | -1.19 | -53.50 | -0.09 |
| 1GC1 | C:63(ASP->ALA) | -3.49 | -2.69 | -3.92 | -0.32 |
| 1GC1 | C:64(GLN->ALA) | 1.84 | 4.97 | 0.53 | 0.44 |
| 1GC1 | C:66(ASN->ALA) | -0.20 | -0.04 | -0.09 | -0.03 |
| 1GC1 | C:72(LYS->ALA) | -0.77 | -0.44 | -0.53 | -0.02 |
| 1GC1 | C:73(ASN->ALA) | 0.01 | -0.01 | -0.02 | -0.11 |
| 1GC1 | C:75(LYS->ALA) | -0.47 | -0.53 | -0.52 | 0.16 |
| 1GC1 | C:77(GLU->ALA) | 0.30 | 0.45 | 0.43 | 0.56 |
| 1GC1 | C:81(THR->ALA) | 0.01 | 0.02 | 0.35 | 1.50 |
| 1GC1 | C:85(GLU->ALA) | -1.48 | -1.48 | 0.03 | 1.31 |
| 1GC1 | C:86(VAL->ALA) | -0.19 | -0.11 | -4.61 | -0.07 |
| 1GC1 | C:87(GLU->ALA) | -0.48 | -0.42 | -0.71 | 0.22 |
| 1GC1 | C:88(ASP->ALA) | 0.36 | 0.20 | -1.97 | -0.07 |
| 1GC1 | C:89(GLN->ALA) | 0.16 | 0.11 | 3.96 | 0.17 |
| 1GC1 | C:90(LYS->ALA) | -0.36 | 1.24 | -12.92 | 0.05 |
| 1GC1 | C:91(GLU->ALA) | -0.15 | 0.24 | 0.11 | -0.13 |
| 1GC1 | C:92(GLU->ALA) | 0.42 | 0.39 | -0.04 | 0.02 |
| 1GC1 | C:94(GLN->ALA) | -0.04 | 0.00 | -0.09 | -0.11 |
|  |  |  |  |  |  |
| 1JCK | B:20(THR->ALA) | 5.91 | 4.10 | -4.62 | 1.40 |
| 1JCK | B:23(ASN->ALA) | 6.49 | 6.25 | 16.55 | 2.50 |
| 1JCK | B:26(TYR->ALA) | 5.45 | 3.05 | 13.88 | 1.70 |
| 1JCK | B:60(ASN->ALA) | 0.74 | 1.95 | 6.52 | 1.30 |
| 1JCK | B:90(TYR->ALA) | 3.95 | 3.33 | 4.20 | 2.50 |
| 1JCK | B:91(VAL->ALA) | 5.60 | 5.44 | 10.75 | 2.10 |
| 1JCK | B:102(GLY->ALA) | -2.68 | -2.22 | -3.10 | 0.10 |
| 1JCK | B:103(LYS->ALA) | 1.99 | 1.37 | -10.22 | 0.40 |
| 1JCK | B:176(PHE->ALA) | 2.38 | 2.72 | -0.68 | 1.90 |
| 1JCK | B:210(GLN->ALA) | 5.83 | 5.88 | 6.17 | 2.50 |
|  |  |  |  |  |  |
| 1JRH | I:47(LYN->ALA) | 16.58 | 14.73 | 20.91 | 3.60 |
| 1JRH | I:47(LYN->MET) | 15.90 | 12.53 | 13.68 | 3.30 |
| 1JRH | I:48(ASN->ALA) | -0.37 | -2.16 | -9.66 | -0.30 |
| 1JRH | I:48(ASN->GLN) | 1.11 | -1.36 | 2.58 | 0.40 |
| 1JRH | I:49(TYR->ALA) | 20.30 | 23.35 | 5.01 | 3.40 |
| 1JRH | I:49(TYR->PHE) | 0.36 | 3.36 | 9.81 | 0.80 |
| 1JRH | I:50(GLY->ALA) | 16.73 | 441.87 | 15.80 | 4.50 |
| 1JRH | I:51(VAL->ALA) | 7.50 | 7.39 | 63.26 | 1.90 |
| 1JRH | I:52(LYS->ALA) | 17.53 | 16.77 | 36.02 | 3.00 |
| 1JRH | I:52(LYS->MET) | 7.62 | 6.13 | 24.87 | 5.00 |
| 1JRH | I:53(ASN->ALA) | 13.99 | 13.76 | 24.33 | 3.90 |
| 1JRH | I:54(SER->ALA) | 0.10 | 0.20 | -1.39 | 0.30 |
| 1JRH | I:55(GLU->ALA) | 0.41 | -0.35 | -3.68 | -0.40 |
| 1JRH | I:56(TRP->PHE) | 0.47 | 0.22 | 11.86 | -0.70 |
| 1JRH | I:56(TRP->TYR) | 3.61 | -1.94 | 8.81 | 0.00 |
| 1JRH | I:79(ASN->ALA) | 1.44 | 1.48 | 35.47 | -0.40 |
| 1JRH | I:82(TRP->ALA) | 5.35 | 5.34 | 23.94 | 4.50 |
| 1JRH | I:82(TRP->PHE) | 1.37 | 1.37 | 13.32 | 1.10 |
| 1JRH | I:82(TRP->TYR) | 0.53 | 0.38 | 30.37 | 1.10 |
| 1JRH | I:84(ARG->ALA) | 4.06 | 3.61 | -23.21 | -0.30 |
| 1JRH | I:98(LYS->ALA) | 2.87 | -0.94 | 21.75 | 0.00 |
| 1JRH | L:27(GLU->ALA) | 8.46 | 3.53 | 51.63 | 0.54 |
| 1JRH | L:28(ASP->ALA) | -0.48 | 2.71 | -1.80 | 0.44 |
| 1JRH | L:30(TYR->ALA) | 5.88 | 5.48 | -9.48 | 1.10 |
| 1JRH | L:91(TYR->ALA) | 1.18 | 0.94 | -50.42 | 0.58 |
| 1JRH | L:92(TRP->ALA) | 15.64 | 17.10 | -4.54 | 2.80 |
| 1JRH | L:93(SER->ALA) | -3.69 | -0.76 | 1.64 | -0.65 |
| 1JRH | L:94(THR->ALA) | 2.97 | 3.97 | 4.70 | 0.38 |
| 1JRH | L:96(TRP->ALA) | 6.13 | 5.87 | 7.78 | 1.70 |
| 1JRH | H:32(TYR->ALA) | 1.34 | 5.02 | 18.05 | 1.40 |
| 1JRH | H:52(TRP->ALA) | 6.33 | 6.62 | 9.65 | 2.70 |
| 1JRH | H:53(TRP->ALA) | 5.96 | 6.01 | 0.64 | 2.40 |
| 1JRH | H:54(ASP->ALA) | 7.91 | 8.19 | 26.53 | 1.90 |
| 1JRH | H:55(ASP->ALA) | -0.33 | -0.34 | -0.57 | 1.70 |
| 1JRH | H:56(ASP->ALA) | 3.82 | 3.63 | 19.40 | 1.80 |
| 1JRH | H:58(TYR->ALA) | 8.93 | 8.31 | 24.24 | 1.20 |
| 1JRH | H:95(ARG->ALA) | 9.79 | 5.19 | 25.77 | 0.54 |
| 1JRH | H:98(PHE->ALA) | -0.13 | -0.10 | 1.86 | 0.00 |
| 1JRH | H:99(TYR->ALA) | 13.49 | 12.80 | 13.22 | 1.10 |
| 1JRH | H:100(GLY->ALA) | -1.89 | -1.90 | -3.22 | 1.70 |
|  |  |  |  |  |  |
| 1NMB | L:32(TYR->PHE) | 8.66 | 7.35 | 7.60 | 1.70 |
| 1NMB | L:93(THR->PHE) | 107.32 | 4.59 | 101.90 | 0.10 |
| 1NMB | L:93(THR->TRP) | 132.79 | 181.16 | 99.18 | 0.30 |
| 1NMB | L:94(LEU->VAL) | -0.25 | -0.49 | -0.01 | 0.90 |
| 1NMB | H:56(ASP->ASN) | 4.76 | 2.63 | -1.47 | 2.80 |
| 1NMB | H:56(ASP->GLU) | 0.24 | 7.77 | 4.90 | 2.40 |
| 1NMB | H:99(TYR->ALA) | 3.95 | 4.01 | 4.96 | 1.50 |
| 1NMB | H:100A(TYR->PHE) | -0.20 | -0.86 | 0.93 | 0.50 |
|  |  |  |  |  |  |
| 1VFB | A:30(HIS->ALA) | 1.34 | -0.12 | 1.06 | 0.80 |
| 1VFB | A:32(TYR->ALA) | 10.48 | 11.99 | 16.61 | 1.30 |
| 1VFB | A:49(TYR->ALA) | 0.55 | 1.57 | -17.88 | 0.80 |
| 1VFB | A:50(TYR->ALA) | 8.72 | 3.99 | 5.63 | 0.40 |
| 1VFB | A:53(THR->ALA) | 6.01 | 5.57 | 0.73 | -0.23 |
| 1VFB | A:92(TRP->ALA) | 13.55 | 13.22 | 11.82 | 1.71 |
| 1VFB | A:93(SER->ALA) | 0.32 | -0.42 | -0.57 | 0.11 |
| 1VFB | B:30(THR->ALA) | 0.10 | 0.02 | 0.05 | 0.09 |
| 1VFB | B:32(TYR->ALA) | 3.53 | 2.54 | 3.17 | 0.50 |
| 1VFB | B:52(TRP->ALA) | 8.44 | 8.61 | 8.95 | 1.23 |
| 1VFB | B:54(ASP->ALA) | 3.43 | 2.98 | 2.62 | 1.95 |
| 1VFB | B:56(ASN->ALA) | -0.02 | 0.16 | -6.89 | 0.20 |
| 1VFB | B:58(ASP->ALA) | -0.76 | -0.90 | 1.86 | -0.20 |
| 1VFB | B:98(GLU->ALA) | -0.74 | -0.85 | -1.32 | 1.10 |
| 1VFB | B:99(ARG->ALA) | 1.65 | 1.85 | -2.96 | 0.47 |
| 1VFB | B:100(ASP->ALA) | 9.73 | 5.68 | 11.85 | 3.10 |
| 1VFB | B:101(TYR->ALA) | 9.60 | 9.77 | -5.11 | 4.00 |
| 1VFB | C:18(ASP->ALA) | 1.20 | 0.99 | -6.13 | 0.30 |
| 1VFB | C:19(ASN->ALA) | 6.18 | 0.93 | 0.48 | 0.30 |
| 1VFB | C:23(TYR->ALA) | 0.34 | -0.20 | -0.89 | 0.40 |
| 1VFB | C:24(SER->ALA) | 2.41 | 4.32 | -3.58 | 0.80 |
| 1VFB | C:116(LYS->ALA) | 3.01 | 3.53 | 1.39 | 0.70 |
| 1VFB | C:118(THR->ALA) | 0.97 | 3.01 | 2.26 | 0.80 |
| 1VFB | C:119(ASP->ALA) | 3.08 | 3.30 | 11.17 | 1.00 |
| 1VFB | C:120(VAL->ALA) | 1.38 | 1.20 | 1.14 | 0.90 |
| 1VFB | C:121(GLN->ALA) | 22.61 | 24.56 | 19.11 | 2.90 |
| 1VFB | C:124(ILE->ALA) | 2.14 | 0.53 | -3.60 | 1.20 |
| 1VFB | C:125(ARG->ALA) | 11.49 | 10.97 | 19.74 | 1.80 |
| 1VFB | C:129(LEU->ALA) | 0.50 | -0.05 | -2.37 | 0.20 |
|  |  |  |  |  |  |
| 3HFM | Y:15(HIS->ALA) | -0.53 | 0.17 | -0.19 | -0.44 |
| 3HFM | Y:20(TYR->ALA) | 3.59 | 3.55 | 35.16 | 4.20 |
| 3HFM | Y:21(ARG->ALA) | 14.07 | 10.69 | 8.60 | 0.85 |
| 3HFM | Y:63(TRP->ALA) | -0.05 | 0.46 | 36.02 | 0.31 |
| 3HFM | Y:73(ARG->ALA) | 12.58 | 12.11 | -4.20 | -0.33 |
| 3HFM | Y:75(LEU->ALA) | 4.75 | -4.94 | 11.82 | 0.69 |
| 3HFM | Y:89(THR->ALA) | 0.92 | 0.71 | 2.85 | 0.00 |
| 3HFM | Y:93(ASN->ALA) | 6.03 | 8.49 | 4.74 | 0.21 |
| 3HFM | Y:96(LYS->ALA) | 12.12 | 8.49 | -3.29 | 6.30 |
| 3HFM | Y:97(LYS->ALA) | 11.69 | 7.31 | 7.80 | 5.50 |
| 3HFM | Y:98(ILE->ALA) | -0.27 | -0.23 | -155.43 | 0.00 |
| 3HFM | Y:100(SER->ALA) | 1.14 | 0.55 | -3.35 | 0.26 |
| 3HFM | Y:101(ASP->ALA) | -6.42 | -14.18 | -11.19 | 0.94 |
| 3HFM | H:53(TYR->ALA) | 5.14 | 9.93 | 14.51 | 3.29 |
| 3HFM | H:58(TYR->ALA) | 8.37 | 8.72 | 1.68 | 1.70 |
| 3HFM | H:101(ASP->ALA) | 0.36 | 0.47 | 2.98 | 3.75 |
|  |  |  |  |  |  |
| 1C4Z | A:637(GLN->TRP) | -0.68 | 1.11 | 1.50 | -0.64 |
| 1C4Z | A:641(ASP->TYR) | 49.47 | 2.74 | 4.40 | -1.10 |
| 1C4Z | A:641(ASP->TRP) | -0.42 | 5.77 | 10.45 | -0.86 |
| 1C4Z | D:63(PHE->TRP) | -3.41 | -1.83 | -2.56 | 0.79 |
| 1C4Z | D:64(LYS->LEU) | 0.64 | -4.23 | -2.75 | -0.54 |
| 1C4Z | D:98(ALA->TRP) | 148.19 | -7.85 | -8.69 | -1.90 |
|  |  |  |  |  |  |
| 2OM2 | A:116(GLU->LEU) | -9.18 | -7.49 | -7.52 | -1.05 |
| 2OM2 | A:147(GLN->LEU) | -0.57 | -1.18 | 3.33 | -0.84 |
| 2OM2 | B:508(GLN->LEU) | 1.39 | -3.78 | -4.05 | 3.17 |
| 2OM2 | B:518(LEU->TYR) | 0.06 | 4.39 | 2.57 | 0.07 |
| 2OM2 | B:525(VAL->TRP) | -1.82 | -1.62 | -1.23 | -1.16 |
| 2OM2 | B:529(PHE->TRP) | -5.19 | 8.02 | -0.81 | -1.65 |
